# Adenovirus Fibers as Ultra-Stable Vehicles for Intracellular Nanoparticle and Protein Delivery

**DOI:** 10.3390/biom12020308

**Published:** 2022-02-15

**Authors:** Chrysoula Kokotidou, Fani Tsitouroudi, Georgios Nistikakis, Marita Vasila, Katerina Papanikolopoulou, Androniki Kretsovali, Anna Mitraki

**Affiliations:** 1 Department of Materials Science and Technology, University of Crete, 70013 Heraklion, Crete, Greece; chkokoti@hotmail.com (C.K.); nistikakisgiorgos@hotmail.com (G.N.); maritavsl@gmail.com (M.V.); papanikolopoulou@fleming.gr (K.P.); 2 Institute of Electronic Structure and Laser (IESL), FORTH, 70013 Heraklion, Crete, Greece; ftsitouroudi@yahoo.com; 3 Institute of Molecular Biology and Biotechnology (IMBB), FORTH, 70013 Heraklion, Crete, Greece; kretsova@imbb.forth.gr

**Keywords:** adenovirus, protein fiber, fibritin foldon, biotinylation, nanorod, vector, delivery, stability, nanoparticles

## Abstract

Protein-based carriers are promising vehicles for the intracellular delivery of therapeutics. In this study, we designed and studied adenovirus protein fiber constructs with potential applications as carriers for the delivery of protein and nanoparticle cargoes. We used as a basic structural framework the fibrous shaft segment of the adenovirus fiber protein comprising of residues 61–392, connected to the fibritin foldon trimerization motif at the C-terminal end. A fourteen-amino-acid biotinylation sequence was inserted immediately after the N-terminal, His-tagged end of the construct in order to enable the attachment of a biotin moiety in vivo. We report herein that this His-tag biotinylated construct folds into thermally and protease-stable fibrous nanorods that can be internalized into cells and are not cytotoxic. Moreover, they can bind to proteins and nanoparticles through the biotin–streptavidin interaction and mediate their delivery to cells. We demonstrate that streptavidin-conjugated gold nanoparticles can be transported into NIH3T3 fibroblast and HeLa cancer cell lines. Furthermore, two streptavidin-conjugated model proteins, alkaline phosphatase and horseradish peroxidase can be delivered into the cell cytoplasm in their enzymatically active form. This work is aimed at establishing the proof-of-principle for the rational engineering of diverse functionalities onto the initial protein structural framework and the use of adenovirus fiber-based proteins as nanorods for the delivery of nanoparticles and model proteins. These constructs could constitute a stepping stone for the development of multifunctional and modular fibrous nanorod platforms that can be tailored to applications at the sequence level.

## 1. Introduction

Intracellular delivery of proteins or nucleic acids through a protein carrier is a potentially game-changing strategy for therapeutics. Common challenges that have to be addressed for this approach are the effective uptake of the protein–cargo carrier into the cell, the vector stability, the intracellular release of the cargo and the risks associated with the vector’s eventual cytotoxicity. To develop a delivery agent for therapeutics, several important steps should be taken into account, such as an initial cell-surface binding followed by internalization, efficient release from the endosome into the cytosol and intracellular trafficking towards the compartment of interest, for example, the nucleus [1]. 

Adenovirus virions are the most commonly used vehicles that have been developed so far for gene therapy [2,3,4] or cancer vaccine purposes [5] and more recently as vaccine platforms in the context of the SARS-CoV-2 pandemic [6]. The adenoviral virions are built from three major structural proteins; 240 copies of the hexon trimeric protein form the icosahedral capsid that also comprises 12 pentons located at the capsid vertices. Each penton consists of five copies of the penton protein connected to a fiber protein [7]. Of particular interest is the ability of co-expressed penton plus fiber proteins to self-assemble into dodecahedral particles that could be used as “minimal” vectors for gene therapy [8]. These viral-like particles (VLP)s can also be used to deliver drugs [9], enzymes or large proteins such as immunoglobulins [10] in cells [11]. Engineered adenovirus capsid proteins, such as penton base capsomers of Ad2 and Ad5, have been shown to facilitate the entry of various membrane-impenetrable molecules including genes [12], siRNA [13] and corroles [14]. Moreover, the adenoviral hexon protein, when fused into a polyethylenimine-plasmid complex, can enhance the nuclear delivery of DNA due to its nuclear homing capability [15]. 

The number of reports on the intracellular uptake of adenovirus fiber proteins is considerably smaller than the ones focusing on virions, penton VLPs or penton capsomers. The soluble full-length fiber of Ad5 demonstrated high uptake levels into mammalian cells in the absence of the entire viral capsid, utilizing an actin-mediated and temperature-independent entry pathway. A potential route suggested could be through the heparan sulfate glycosaminoglycans (HS-GAGs) receptors found in cell lines. The fiber protein can assemble into a complex with protamine-condensed plasmid DNA and facilitate gene transfer [16]. Given the aforementioned report and the exceptional stability of the fiber proteins, we deemed that they merit further investigation as stable potential cargo carriers for cellular delivery. 

Adenovirus type 2 fibers are homo-trimers composed of an N-terminal domain attached to the viral capsid, a central fibrous shaft and a C-terminal domain that binds to the cell receptor, namely the Coxsackie and Adenovirus Receptor, termed CAR [17,18,19]. The globular C-terminal “head” or “knob” domain is necessary for the trimerization of the fiber and might act as a registration signal that directs its correct folding and assembly [20,21,22]. Synthetic fiber shaft peptides corresponding to shaft sequences do not fold and assemble into the native shaft fold in isolation; instead, the peptides self-assemble into amyloid-type fibrils [23,24,25]. The shaft segment adopts a trimeric, highly intertwined fold termed the “triple beta-spiral” that conveys onto the fiber protein its exceptional stability [26,27]. The protein is resistant to SDS, temperature, proteases and denaturants [22].

For gene therapy and delivery applications, the targeting of specific cell types is highly desirable. To target vectors onto specific cell types except for the CAR receptor, the knob CAR-binding domain should be substituted with other cell-targeting motifs. However, by removing the knob, the fiber loses its trimerization ability, and an alternative trimerization motif has to be used. Well-known trimerization motifs include triple-coiled coils [28] and the beta-hairpin trimerization motif from phage T4 fibritin [29]. Recombinant adenovirus virions have been previously reported [30], where the entire knob domain of the Ad5 fiber was deleted and replaced by a helical trimerization motif from MoMuLV envelope glycoprotein, a Myc-epitope and a 6 x-His-tag. In the context of these virions, knobless recombinant Ad5 fibers were capable of efficient trimerization and internalization through their His-tag interaction with an anti-His single-chain antibody variant displayed on the cell surface. Bifunctional adapter molecules have been engineered that enable the targeting of the “mosaic” adenovirus fiber to alternative cellular receptors distinct from CAR. A metabolically biotinylated fiber mosaic Ad was fabricated through the incorporation of a biotin acceptor peptide in the sequence of the fiber protein. The hybrid protein containing a luciferase-expressing gene was effectively complexed with an epidermal growth factor (EGF)–streptavidin. The fiber mosaic virion was then retargeted to EGF receptor (EGFR)-expressing cells and exhibited increased infectivity [31].

In the context of structural studies, fiber constructs have been previously designed to comprise a stable shaft segment (residues 319–392) N-terminally fused to the small non-cell targeting trimerization domain, the fibritin foldon, with or without the natural linker that connects the shaft to the globular head [32]. The resulting “shortened” construct comprised four shaft repeats plus the foldon domain. The shaft repeats adopted their correct folding and trimerization ability into nanorods, as was verified by solving the crystal structure of the chimeric proteins [33]. Moreover, they were found to be stable and could withstand extreme conditions, such as SDS, temperature and digestion by proteases.

In the present study, we aimed to design and study a series of almost full-length fiber constructs destined for potential application as protein carriers for intracellular delivery. The approach is again based on the principle of replacing the globular head with the foldon trimerization motif [33], thus removing the CAR receptor specificity. Hybrid fiber constructs were fabricated comprising of a much longer shaft segment, Met 61-Gly 392 with or without the natural linker sequence in addition to the foldon trimerization motif. 

**Scheme 1:**
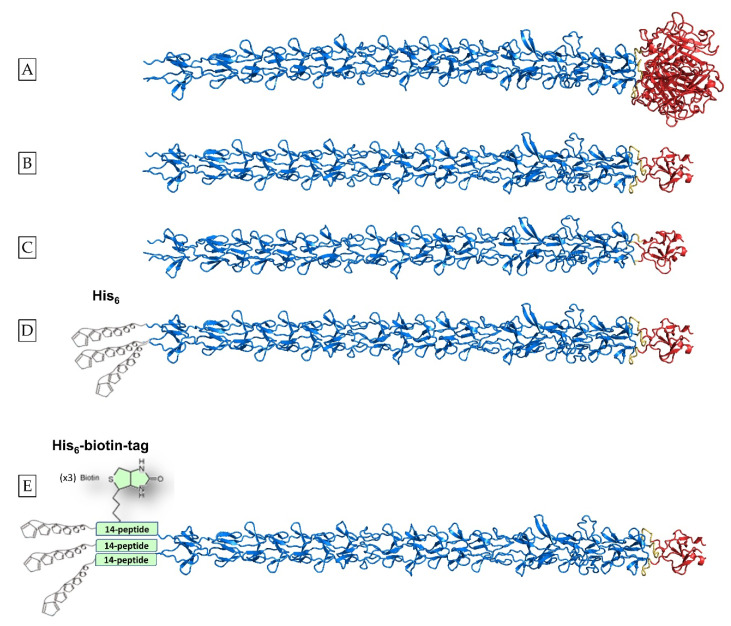
List of designer proteins studied. (**A**) Structure of adenovirus type 2 fiber residues 61–582 generated with the open source AlphaFold-Multimer code [36]. Residues belonging to the shaft domain (Met61-Gly392) are shown in marine blue and residues belonging to the globular head domain (Leu399-Glu582) are shown in red. The natural linker that connects the two domains consists of residues 393–398 (Asn-Lys-Asn-Asp-Asp-Lys) and is shown in yellow. (**B**) Schematic representation of the chimeric protein that comprises the fibritin foldon domain (fibritin residues Gly457–Leu483, shown in red) fused to the C-terminus of the shaft domain (shown in marine blue) with a linker containing the natural linker plus two connecting residues (Asn-Lys-Asn-Asp-Asp-Lys-Gly-Ser; shown in yellow) between the two domains (hereinafter referred to as *protein Linker [L]*) (**C**) without the use of the natural linker (i.e., only the connecting Gly-Ser residues) between the two domains (hereinafter referred to as protein *no Linker [NoL]*). (**D**) Schematic representation of the construct comprising shaft residues 61–392 connected to the fibritin foldon domain with the Asn-Lys-Asn-Asp-Asp-Lys-Gly-Ser linker, plus a hexa-histidine tag at the N-terminal end, hereinafter referred to as *Linker-His [L-H].* (**E**) Schematic representation of the protein hereinafter referred to as *Linker-His-Biotin [LHB].* Shaft residues 61–392 are connected to the fibritin foldon with the Asn-Lys-Asn-Asp-Asp-Lys-Gly-Ser linker; between the His-tag located to the N-terminal end and the first shaft domain amino acid, a 14-amino acid biotinylation site is inserted. The crystal structure of residues 319–582 of the native adenovirus fiber has been solved in [27], PDB code 1 QIU; the crystal structures of shaft residues 319–392 fused to the T4 fibritin foldon domain were also solved in two forms [33] with (PDB code 1V1I) and without the natural linker sequence (PDB code 1V1H). The hexa-histidine tag and the biotin molecule are not drawn to scale compared to the protein structures. Courtesy of Dr. Mark van Raaij, CSIC Madrid, and Dr. Roberto MARABINI, Universitat Autonoma de Madrid.

The first generation of constructs was engineered as follows: a foldon domain was placed at the C-terminus to enable correct trimerization and a 6x His-tag for purification purposes was placed at the N-terminus. A second generation included an additional 14-peptide following the His-tag, which acts as a docking station and enables the binding of biotinylated molecules. The protein was overexpressed along with a biotin ligase enzyme with the addition of both IPTG and biotin for in vivo biotinylation. Protein biotinylation in vivo can be achieved by co-expressing in bacterial cells the protein of interest fused to a 14-amino-acid biotinylation sequence, (G-L-N-D-I-F-E-A-Q-K-I-E-W-H) and the BirA ligase. The BirA ligase specifically biotinylates the lysine side-chain of the biotinylation sequence by joining it with the carboxyl group of the biotin molecule [34,35]. The constructs designed and studied are summarized in Scheme 1.

The constructs were evaluated for their expression and production in *E. coli* cells and ability to fold into a trimeric, rod-like conformation. They were subsequently studied for their stability in regard to temperature and protease digestion following their purification. 

We report below that the His-tag conveys stability to the initial constructs; additionally, the His-tag biotinylated construct folds into thermally and protease-stable fibrous nanorods that can be internalized into mammalian cells and are not cytotoxic. Moreover, they can bind to proteins and nanoparticles through the biotin–avidin interaction and mediate their delivery to cells. We discuss the potential implications for their use as stable delivery vehicles.

## 2. Materials and Methods

### 2.1. Chemical Reagents

Chemical reagents and buffers were purchased from Sigma-Aldrich and Biotin Coated Plates and SuperSignal WestPico Plus chemiluminescent substrate were purchased from Thermo-Scientific. Q-Sepharose and Ni-NTA Sepharose columns were purchased from GE Healthcare. Carbon/Formvar electron microscopy copper grids were purchased from Agar Scientific. AlexaFluor594 FluoroNanogold-Streptavidin was purchased from Nanoprobes.

### 2.2. Cell Cultures

HeLa (human cervical cancer cells) and NIH3T3 (mouse fibroblast cells) were from the cell bank at the Institute of Molecular Biology and Biotechnology (IMBB), FORTH. Cells were cultured at 37 °C in a 5% humidified CO_2_ incubator in Dulbecco′s Modified Eagle’s–Medium (DMEM) growth medium (pH 7.4) from Gibco (Billings, MT, USA) supplemented with 10% Fetal Bovine Serum (FBS) purchased from Gibco and 50 μg·mL^−1^ gentamycin (Applichem, Darmstadt, Germany). Cell fluorescent dyes and NHS-Fluorescein were purchased from Thermo-Scientific, Waltham, MA, USA and nuclear dye DAPI was purchased from Molecular Probes (Eugene, Oregon). 

### 2.3. Antibodies and Plasmids

Penta-His Antibody BSA-free was from Qiagen (Hilden, Germany) and secondary antibody anti-mouse IgG-alkaline phosphatase was purchased from Sigma-Aldrich (St. Louis, MO, USA). Streptavidin conjugated with alkaline phosphatase and streptavidin conjugated with HRP were from Merck (Darmstadt, Germany). 

### 2.4. Molecular Cloning

The pET28a plasmid used was from Novagen (Darmstadt, Germany). Deep Vent polymerase and restriction enzymes used for cloning were purchased from New England Biolabs (Ipswich, MA, USA).

### 2.5. Design of the Chimeric DNA Constructs

The NoLinker and Linker protein constructs were fabricated with molecular cloning techniques using as template the pT7.7 vector containing the foldon trimerization domain sequence (Gly457–Leu483), as previously described [33]. For the NoLinker protein, the DNA sequence corresponding to shaft residues Met61-Gly 392 was amplified with primers 5′-GACACCTCCCACCATATGCTTGCGCTTAAA-3′ and 5′-GGTAAGTTTGTCATCATTGGATCCTCCTATTGTAAT-3′ and inserted between the Nde*I* and Bam*HI* of the vector. For the Linker protein, where the shaft Met61- Gly 392 and the foldon sequences are connected with the natural fiber linker residues Asn393–Lys398 [32], the following primers were used: 5′-GACACCTCCCACCATATGCTTGCGCTTAAA-3′ and 5′-TTGTCCACAGGGATCCTTTGTCATCATTTTTGT-3′. For both constructs, two extra residues (Gly- Ser) were used as a result of the cloning strategy. The chimeric protein constructs Linker-His [L-H] and Linker-His-Biotin [LHB] were generated using the following primers: Forward: 5′-GGCCGAATTCATGCTTGCGCTT-3′ and Reverse: 5′-GCCCCTCGAGTCTATCCTATTGTAATGGC-3′. [L-H] and [LHB] were digested with the enzymes SmaI/ClaI and EcoRI/XhoI accordingly before being ligated with the pET28a vectors.

The L-H encoding sequence was subcloned in the plasmid vector pET28a (+) in order to be in frame with the N-terminally located His-tag sequence. Plasmid pET28a (+) was modified for the addition of the biotinylation encoding site after the N-terminal sequence that encodes the 6 × His-tag sequence, and the new plasmid generated was termed pET28a-bio. The DNA fragment encoding the biotinylation site was amplified with Polymerase Chain Reaction with the use of the forward primer (FW): 5′-GGCGCATATGTCCGGCCTG-3′ and reverse primer (RV): 5′-GCGCGAATTCTTCGTGCCA-3′. The amplified biotinylation fragment and the pET28a plasmid were double digested with the NdeI and EcoRI restriction enzymes. The sequence encoding the Linker protein was finally subcloned into the pET28a-bio plasmid as described above.

### 2.6. Recombinant Protein Expression and In-Vivo Biotinylation of the Chimeric Proteins

The plasmid vectors containing the gene of interest were transformed into BL21 (DE3) *E. coli* cells. The vector containing the LHB protein gene was co-transformed into *E. coli* cells with the pBirAcm vector encoding BirA Ligase in order to catalyze biotin binding to the translated proteins. The transformed cell cultures of the *E. coli* strain BL21 (DE3) carrying the respective vectors were grown at 37 °C in 1 L of LB (Luria Bertani) medium with the corresponding antibiotics (kanamycin for PET28 and chloramphenicol for pBirA plasmid) until the culture density reached an OD600 value of 0.5–0.6. Protein overexpression was induced by 1 mM IPTG plus 1.2 mg/mL biotin dissolved in 10 mL of 10 mM bicine buffer pH: 8 in the case of the LHB construct when biotinylation was desired. Cell cultures were incubated for 4 h at 37 °C aerated by vigorous shaking at 250 rpm. Samples were collected before and after IPTG induction. Cells were harvested by cell culture centrifugation at 6000 rpm for 15 min. 

### 2.7. Inclusion Bodies Lysis and Purification of the Recombinant Proteins

The cell pellet was resuspended in 20 mL of lysis buffer containing 20 mM Tris-HCl pH: 9, 100 mM NaCl, 2% Triton, 1× cOmplete™ Protease Inhibitor Cocktail solution, 1 mM EDTA and 5% glycerol. The bacterial cell lysis was performed by three freeze–thaw cycles at −80 °C and a subsequent submersion in a 37 °C water bath. Addition of 2 mg/mL lysozyme at a final concentration with 1 h incubation at 37 °C followed with subsequent three freeze–thaw cycles. The cell suspension was incubated for 30 min at room temperature while shaking with DNase I at a final concentration of 100 μg/mL and 100 mM MgSO_4_. The cell suspension was finally passed through a French press at 1000 psi in order to achieve the maximum lysis of any remaining intact cells.

The majority of the expressed protein was stored in the inclusion body fraction. The pellet of inclusion bodies after lysis was washed under shaking for 10 min with urea 1 M, then Triton 1% and 20 mM Tris-HCl pH: 9, followed by centrifugation in order to remove non-specifically bound impurities. The resultant pellet was resuspended at the initial volume in 6 M urea, 20 mM Tris-HCl pH 9, NaCl 0.5 M, Imidazole 10 mM resuspension buffer and was stirred overnight. The remaining insoluble products were subsequently separated by centrifugation at 7000 rpm for 30 min and discarded. The proteins were purified from the cell lysate by Ni-NTA affinity chromatography column. 2 mL of bulk Ni-NTA beads was used as stationary phase. The beads were deposited in a gravity flow column and the supernatant was passed twice. Then, the beads were washed with 8 volumes of two washing buffers with increasing imidazole concentration (10 and 30 mM). The protein was eluted with increasing imidazole concentration solutions (50, 75, 100, 150, 250 and 500 mM). Elution and washing buffers also contained 0.5 M NaCl to reduce non-specific binding of proteins. 

### 2.8. Protein Refolding-Dialysis

Following purification, the elution fractions containing the protein of interest were pooled and sealed in a 10–14 kDa molecular weight cut-off dialysis tubing cellulose membrane. The membrane was transferred to a beaker containing 2 L of the desired exchange buffer. Phosphate buffer 20 mM with 0.2 M NaCl was used for the further labeling of the protein with NHS-fluorescein. Tris-HCl pH: 9, 0.2 M NaCl and 5% glycerol were used for all the other assays. In order to achieve exhaustive dialysis, the membrane was immersed initially into the dialysis buffer for 1 h and subsequently relocated into a fresh dialysis buffer and left for buffer exchange overnight at 4 °C. The dialyzed samples were concentrated using an Amicon centrifugal filter (30 kDa molecular weight cut-off) in order to achieve increase in protein concentration. 

### 2.9. Western Blot

The purified proteins were analyzed by SDS-PAGE and Western Blot in order to detect the successful incorporation of His-tag sequences and biotin and for the distinction of the potential degradation products. For His-tag, the mouse Penta-His Antibody BSA-free (Qiagen) was used as primary antibody and for the biotin molecule, streptavidin conjugated with alkaline phosphatase (Sigma-Aldrich, St. Louis, MO, USA) was solely used for detection. Briefly, purified proteins were separated in a 7.5% SDS-PAGE and transferred to PVDF membranes. The membrane was blocked by mild shaking in 5% *w*/*v* skimmed milk in 1× phosphate-buffered saline (PBS) for 1 h at room temperature. After blocking, the PBS-milk was removed and the membrane was soaked in a solution of PBS-milk containing the primary antibody diluted 1:2000 and incubated overnight at 4 °C under mild shaking. After 3 washes by shaking for 15 min in a solution of 1× PBS with 0.04% Tween 20, the blots were incubated in PBS-milk containing the secondary antibody (anti-mouse IgG-alkaline phosphatase) diluted 20,000-fold for 2 h at room temperature under mild shaking. Three washes followed and the protein bands were visualized with the addition of 176 μL/10 mL NBT-BCIP substrate (Sigma) in alkaline phosphatase buffer (0.1 M Tris-HCl pH: 9, 0.1 M NaCl, 5 mM MgCl_2_). The same methodology was used for the detection of the fibritin foldon motif. The T4 fibritin anti-wac Antibody (Cusabio) was used as the primary antibody at the ratio of 1:1000 and anti-rabbit IgG conjugated with alkaline phosphatase as a secondary antibody at a ratio of 1:20,000. For biotin detection, the blot was incubated with streptavidin conjugated with alkaline phosphatase for 1 h at room temperature and was subsequently washed with PBS-Tween20. Following a rapid wash with alkaline phosphatase buffer, the chemiluminescent detection was performed with the addition of the BCIP-NBT substrate. The reaction was stopped by rinsing and substituting the reaction buffer with distilled water. 

### 2.10. TEM Observation

Transmission Electron Microscopy after negative staining was used to observe the conformation of the LHB protein following purification and refolding. A protein sample solution (concentration 20 μg/mL) of 8 μL was deposited onto a carbon/formvar-covered copper grid. The sample solution was left onto the grid for 2 min and the excess solution was removed using a Whatman filter paper. Subsequently, 8 μL of 1% Uranyl acetate was applied, left for 2 min and the excess solution was removed using a filter paper. Samples were observed in a JEOL JEM 2100 High-Resolution microscope, operating at 200 kV (University of Crete, Biology Department).

### 2.11. Thermostability Evaluation with SDS-PAGE and Coomassie Blue Staining

10 μL of the LHB protein at a 0.4 mg/mL concentration that was previously stored at 4 °C was mixed with 5 μL sample buffer containing 0.1% SDS. The sample was immediately frozen at −20 °C. 10 μL of LHB protein of the same concentration was left at RT for 30 min and subsequently mixed with 5 μL sample buffer containing 0.1% SDS. The sample was also immediately frozen at −20 °C. 10 μL of the LHB protein at a 0.3 mg/mL concentration that was previously stored at 4 °C was added into a preheated Eppendorf tube of 1.5 mL capacity which was placed in a heat-block in order to equilibrate in the desired temperature. Starting from 37 °C, the aforementioned procedure was repeated while the temperature was increased reaching specific temperature points every 30 min. The protein sample was incubated at the desired temperature for 30 min. After the 30-min incubation, 5 μL sample buffer (0.1% SDS) was added and the sample was frozen at −20 °C. Temperature points for 30 min incubation each were the following: 4, 25, 37, 50, 60, 70, 80, 100 °C. The sample at 100 °C was prepared twice in two different tubes. In the first, the sample buffer added contained 0.1% SDS and in the other, it was 2%. The samples were loaded on a 7.5% SDS-PAGE gel and electrophoresed in the cold room at a constant voltage of 150 V; they were subsequently stained with Coomassie Brilliant Blue R-250 for the detection of the folded and unfolded protein samples. 

### 2.12. Chymotrypsin Digestion 

The susceptibility of the LHB protein to proteases was assessed with the chymotrypsin digestion assay. Chymotrypsin was incubated with the LHB protein (conc: 0.2 mg/mL) at protease-to-protein molar ratios of 1:200 and 1:50, for 10 min at RT. As a control, an untreated protein sample was used. The activity of the protease was quenched with the addition of 2% SDS sample buffer and immediate freezing at −20 °C. The samples were loaded on a 7.5% SDS-PAGE gel and electrophoresed at 180 V. Thawed protein samples to be electrophoresed were either unboiled to assess the digestion effect on the trimeric form of the protein or heated at 100 °C for 5 min before SDS-PAGE to evaluate the digestion effect on the monomer form. Unboiled digested samples were electrophoresed in the cold room whereas the boiled samples were at RT conditions. Western Blot analysis was performed as described above, using the anti-His and the foldon wac antibody to detect the terminal end(s) where the protease starts trimming.

### 2.13. Protein Binding with NHS-Fluorescein and Nanoparticles Conjugated with Fluorophores 

0.2 mg/mL of protein in 20 mM phosphate buffer pH: 9, NaCl: 0.2 M was conjugated with a 15-fold molar excess of NHS-Fluorescein (N-Hydroxysuccinimide (NHS)-ester Fluorescein) and incubated for 2 h at room temperature. The unbound fluorescein was removed by exhaustive overnight dialysis in 20 mM phosphate buffer pH: 9, NaCl: 0.2 M and the protein was stored at 4 °C. 4-fold of the biotinylated and NHS-fluorescein labeled LHB protein (80 μg/mL) was incubated with the labeled NPs for 1 h at room temperature. The excess of the unbound streptavidin fluorescent NPs was removed by incubating the mixture into a biotin-coated plate (Thermo-Scientific). LHB labeled with NHS-fluorescein was used for the binding assay of the streptavidin-Alexa-NP for confocal microscopy co-localization observations.

### 2.14. LHB Protein Linkage with Active Enzymes through the Biotin-Streptavidin Bond

The biotinylated LHB protein is interacting with the streptavidin–alkaline phosphatase (strept-AP) and the streptavidin–horseradish peroxidase (strept-HRP) conjugates through the biotin–streptavidin non-covalent bond. Samples of various ratios of LHB:strept-AP or LHB:strept-HRP (1:3, 1:1, 3:1) were incubated for 2 h at RT. LHB had a fixed concentration at 0.4 mg/mL. The optimal binding ability was verified in a 1% agarose gel with the addition of a native protein sample buffer (without SDS). The gel was run in the cold room at 65 V.

### 2.15. Cell Internalization and Trafficking of the Proteins and Protein-NP Conjugates

NIH3T3 fibroblast and HeLa cell lines were cultured at 37 °C, 5% CO_2_ in DMEM supplemented with 10% fetal bovine serum and 50 μg/mL gentamycin. 8 × 10^4^ HeLa or NIH3T3 fibroblast cells were seeded for 24 h in a 24-well plate after addition of a 13 mm TC Coverslip at the bottom of the well. The culture medium (DMEM) was replaced and a 50 μL protein sample containing 50 μg/mL of the fluorescein labeled protein or protein-NPs conjugate, diluted in 0.5 mL DMEM, was added for overnight incubation. Culture media was aspirated, and the cells were carefully washed two times with PBS 1×. The cells were fixed with 4% formaldehyde for 15 min, washed twice with PBS 1× and then the coverslip was mounted on a microscope coverslip containing a drop of the DAPI nuclear staining dye. The internalization and subcellular localization of the proteins was assessed with a Leica SP8 inverted confocal microscope at excitation/emission wavelengths of 488/520 nm for the Fluorescein–protein conjugate, excitation/emission of 360/460 nm for DAPI nucleus stain and excitation/emission of 594/617 nm for the Alexafluor594-bound NPs.

### 2.16. MTT Cell Proliferation Assay

The cell viability in the presence of the LHB protein only was studied by monitoring the conversion of Thiazolyl Blue Tetrazolium Bromide reagent (MTT) into formazan by the mitochondrial dehydrogenases of the living cells. NIH3T3 fibroblast and HeLa cells with concentrations of 7 × 10^3^ cells/well were cultured in a 96 plate for 24 h. Removal of the medium was followed by treatment of the cells with increasing concentrations (10–800 μg per well) of the protein, suspended in a total volume of 200 μL of culture medium. Cells that were not treated with the protein served as control. After 48 h incubation, the medium was carefully removed and replaced with 90 μL of fresh medium and 10 μL of MTT (5 mg/mL) dissolved in PBS 1×. The cells were incubated for 4 h to allow the development of the purple formazan products and the MTT-culture medium was substituted with 100 μL of isopropanol-DMSO 1:1 solution. The formazan crystals were allowed to dissolve for 15 min at 37 °C. The absorbance was measured at 570 nm in a Synergy HTX BioTEK Plate Reader.

### 2.17. Measurement of the Internalization Efficacy and Enzymatic Activity of LHB-Strept-AP

The LHB protein at a concentration of 50 μg/mL was incubated with the streptavidin-Alkaline Phosphatase (AP) conjugate at a 1:1 ratio. The final volume of the mixture was 50 μL in 20 mM Tris-HCl pH: 9, 0.2 M NaCl. After 1-h incubation, the mixture was transferred to a biotin-coated plate for 30 min in order to remove the excess of the streptavidin-AP conjugate. The enzyme-linked LHB was added to overnight precultured NIH3T3 cells (initial cell density 8 × 10^4^/well) in a 24 well plate. HeLA cells were not used for this assay due to the high inherent production of AP in this cell line. The conjugates were incubated with 500 μL of DMEM at 37 °C overnight and the following day, the medium was removed and the cells were washed two times with PBS 1×. The internalization and the enzymatic activity of Alkaline phosphatase were assessed by the addition of 7 μL of the substrate BCIP-NBT in each well containing 300 μL of PBS. The reaction product has a blue color and is insoluble in water, therefore rendering the cells visible with a dark coloring where the product is located. As controls, untreated cells and the streptavidin-AP alone at the same concentration were used. The cells were visualized with an optical microscope. 

### 2.18. Measurement of the Internalization Efficacy and Enzymatic Activity of LHB-Strept-HRP

The LHB–streptavidin–Horseradish peroxidase (LHB-strept-HRP) was formed and incubated with the cells similarly as described for the LHB–streptavidin–Alkaline Phosphatase conjugate. HeLa cancer cells were also used for these assays since there is no inherent production of HRP in this cell line. After 24-h incubation of the conjugates with the cells, the enzymatic activity and internalization efficacy of the LHB conjugate was assessed by 3,3′-diaminobenzidine (DAB) staining and luminescence detection. Untreated cells and streptavidin-HRP alone at the same concentration were used as controls. Each sample and control was assessed in triplicates. For observation using light microscopy, NIH3T3 and HeLa cells were washed twice with PBS to remove excess uninternalized conjugates. 0.1% of Triton X-100 was added for 5 min and the cell washing was followed with PBS prior to DAB staining. HRP activity was detected by the brownish precipitation formed following the addition of 300 μL of 0.05% DAB in the presence of 1 μL H_2_O_2_ (30%). The precipitates were left to form for several hours, and the cells were observed with an optical microscope. Quantitative measurement of the HRP enzymatic activity was performed by luminescence detection. The enzymatic activity and transfer ability of LHB linked with strept–HRP was quantified after the 24 h incubation of the cells with the conjugates. LHB:strept–HRP ratio was 1:1, the total volume of the conjugate was 50 μL and the concentration of LHB was 50 μg/mL. Following incubation, the cells were washed twice with PBS. Subsequently, 300 μL of PBS-EDTA 1 mM were added to the well and the well plate was incubated at 37 °C for 5 min for cell detachment. The cells were detached by intense pipetting from the bottom of the well and transferred to a 1.5-milliliter tube. The procedure was repeated for the complete detachment of the cells. The tubes were centrifuged at 5500 rpm for 5 min. The supernatant was removed and the cells were lysed with 30 μL of lysis buffer (25 mM Tris-Phosphate buffer pH: 7.8, 2 mM EDTA, 10% glycerol, 1% Triton X-100, 2 mM DTT) for 30 min. Luminol substrate and H_2_O_2_ (SuperSignal WestPico Plus chemiluminescent substrate) were added at equal volumes (25 μL) and the solution was immediately transferred to a 96-well plate for measuring the luminescence in a Synergy HTX BioTEK Plate Reader. 

### 2.19. Statistical Analysis 

Statistical analysis was performed using the Student’s *t*-test. Statistical significance was indicated with the value of probability (*p*) (* *p* < 0.05, ** *p* < 0.01, and *** *p* < 0.001).

## 3. Results

### 3.1. Overexpression, Purification and Folding of Linker (L) and No-Linker (No-L) Chimeric Proteins

**Figure 1:**
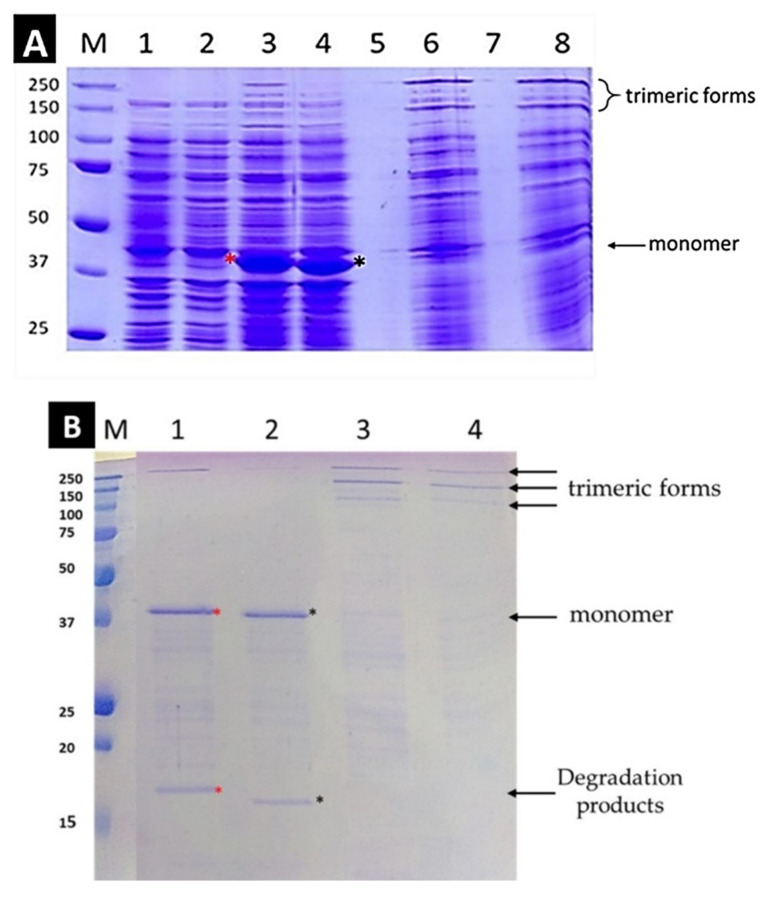
(**A**). Overexpression of chimeric proteins [L] and [NoL] before and after induction with IPTG. SDS-PAGE with a 7.5% gel was performed at RT. Lanes: M: protein markers, 1 + 2: proteins [L] and [NoL], respectively before induction with IPTG, 3 + 4: proteins [L] and [NoL] respectively after 4 h of the induction. The samples are boiled at 100 °C for 5 min before loading. At ~40 kD a discrete band is observed for both proteins corresponding to the molecular mass of the monomer. The red asterisk indicates the band of L which is slightly higher than the band of NoL (black asterisk). The mobility difference of the two bands, which is due to the presence of the linker, is clearly observed. Lanes 5 and 7 are empty. 6 + 8: Protein [L] and [NoL] after 4 h of induction without boiling the sample. (**B**): Refolding of the proteins after Dialysis. Protein [L] (red asterisk) is detected higher than [NoL] (black asterisk). Lanes: M: protein markers; 1: protein [L] dialyzed, boiled; 2: protein [L] dialyzed, unboiled; 3: protein [NoL] dialyzed, boiled; 4: protein [NoL] dialyzed, unboiled. SDS-PAGE with a 12% gel was performed at RT.

The Linker and No-Linker proteins were overexpressed and purified in order to assess their ability to fold and assemble into the stable trimeric form. Bacterial overexpression was performed in BL21 DE3 *E. coli* cells. The constructs could be overexpressed in abundant quantities (Figure 1A). 

The gel electrophoresis of the lysates of the expressed proteins is shown in Figure 1B. The bands observed at ~40 kD in lanes three and four confirm the overexpression of both proteins in abundant quantities. In lanes six and eight, the monomeric bands observed in lanes 3 and 4 disappear and two new bands appear at high molecular mass positions. As previously reported, in the presence of SDS and upon boiling at 100 °C, the full-length native adenovirus fiber starts to unfold from its N-terminus end [22]. This result suggests that the two proteins [L] and [NoL] must adopt a trimeric form and unfold to the monomer form only upon boiling. Therefore, the foldon functions successfully as a trimerization motif for these constructs. 

Due to the abundant expression, the protein tends to aggregate into inclusion bodies (IBs) [37,38], inside the bacterial cytoplasm. After cell lysis, in order to isolate and purify the protein in its native state, inclusion bodies should be washed and further dissociated. In order to wash them, a low concentration of chaotropic agents and mild detergents were used successively to dissolve and easily remove most of the impurities (Figure S3).

To retrieve the proteins in their native form, the inclusion body aggregates formed by the protein should be denatured by urea, purified and refolded after dialysis to remove the denaturing agent. After the dissociation of the IB with 6 M urea containing 20 mM Tris pH: 9, the lysates were loaded into a Q-sepharose column. The elution fractions of the protein were collected with increased concentrations of NaCl (Figure S4). Exhaustive dialysis followed the purification for both proteins in order to completely remove urea and trigger the refolding of the proteins. Excess of protease inhibitors was used in an attempt to inhibit any protein degradation that might occur due to recovery of any protease activities during urea removal. 

The electrophoretic profile of the refolded and purified proteins is shown in Figure 1B. In lanes one and two, below the bands corresponding to the monomeric protein forms at ~40 kD lower molecular mass, bands are detected in the range of 15–20 kDa. The difference in molecular mass between the two constructs is attributed to the presence or absence of the six amino acid natural linker that connects the shaft segment with the trimerization motif; it is noteworthy that the mobility difference is maintained in the lower molecular mass bands. These must correspond to degradation products that can be attributed to protease activity following protein overexpression, cell lysis, and /or refolding steps. In lanes three and four, (non-boiled samples) the monomer bands disappear and slowly migrating bands appear, corresponding to the trimeric full-length protein and its partially unfolded and /or partially digested forms that still remain trimeric. The presence of the most slowly migrating band can be attributed to a partially unfolded and expanded conformation of the full-length protein due to SDS (see section supporting information methods and Figure S1).

### 3.2. Design and Study of the Linker-His (L-H) Protein as a Hybrid Construct with Additional Functionalities

Through the foldon addition at the C-terminus, we achieved overexpression of the protein; however, the production of degradation products could not be avoided even if protease inhibitors were used throughout the protein manipulation. It was deduced that the protein may be affected by proteases and being truncated following the protein overexpression, lysis and/or purification steps. It was previously established that the native full-length fiber starts to unfold from the N-terminal end; the unfolded part becomes susceptible to proteases [22]. We therefore hypothesized that inserting a Histidine–tag at the N-terminus might convey decreased sensitivity to proteases, therefore we proceeded to fabricate the Linker-His (L-H) construct (Scheme 1D). Moreover, His-tag sequences have been previously reported to act as “endosomal escape” factors in mammalian cells [39,40,41]. The imidazole group of histidine, with a pK around six protonates within the endosomes in increasingly acid conditions. This results in endosomal lysis and release of cargo in the cell cytosol, allowing the initial protection and subsequent availability of the protein in the cytosol. This is therefore beneficial for the delivery of protein cargo. Additionally, His-tags have been thoroughly used for creating a complex with nickel ions usually coordinated with Nitro-Triacetic-Acid (NTA) for Ni-NTA affinity purification [41]. Moreover, it was reported that the His-tag tails can mediate the self-assembly of protein nanoparticles used as vehicles in non-viral gene therapy [42].

**Figure 2:**
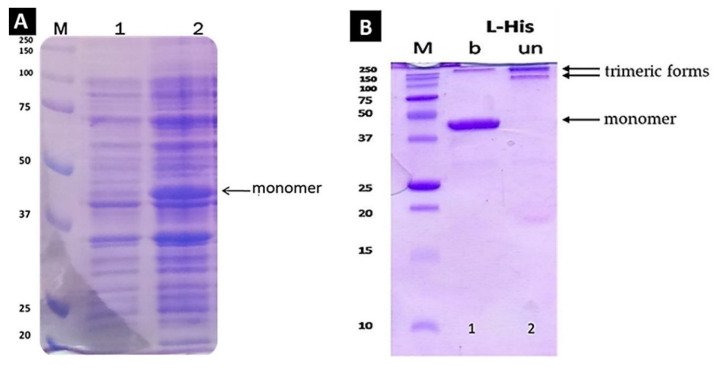
(**A**). Overexpression of the chimeric protein [L-His] before and after induction with IPTG (boiled). SDS-PAGE with a 7.5% gel was performed at RT. Lanes: M: protein markers, 1: protein[L-His] before induction with IPTG, 2: protein after 4 h of induction (boiled sample at 100 °C for 5 min). At ~40 kD a discrete band is observed, confirming the protein overexpression. (**B**) SDS-PAGE of purified Linker-His protein after dialysis. SDS-PAGE with a 12% gel was performed at RT. Lane M, molecular markers; Lane 1, Boiled (b) sample, Lane 2 Unboiled (un) sample. The monomer migrates at 40 kDa after boiling in SDS sample buffer; no degradation products were observed underneath the monomer band.

We opted to continue with the Linker protein, termed L-His, as a template for further cloning, as the natural linker is expected to retain the flexibility of the native protein and convey enhanced stability as previously reported in [33]. The chimeric protein L-His was successfully overexpressed in *E. coli* (Figure 2A) and purified, following the inclusion body dissociation with urea, using a Ni-NTA column. The protein was eluted with an increasing concentration of imidazole at concentrations 50–100 mM. 

A dialysis step followed with the buffer containing Tris 20 mM pH: 9, NaCl 0.2 M and glycerol 5% for the removal of urea, imidazole and high concentration of NaCl salt. Protease inhibitors were also added for protection against possible remaining traces of proteases (Figure 2B). In lane one is distinguished the purified protein at 40 kDa after boiling in SDS loading buffer at 100 °C. No other proteins were detected, confirming the successful purification of the protein. In lane two, where the same protein samples were loaded without boiling, the band at 40 kDa disappears with the concomitant appearance of two slowly migrating bands, thus confirming the successful trimerization of the protein. Moreover, no lower molecular mass bands are observed underneath the boiled samples, (compared to lane 1 in Figure 1B). From these results, it is evident that the chimeric protein Linker-His is able to fold into the trimeric conformation with no proteolytic degradation products. Therefore, the addition of a His-tag at the N-terminus end indeed stabilizes against “frailing” of this end and proteolytic degradation. 

### 3.3. Linker-His-Biotin (LHB) Protein Construct Production and Characterization 

#### 3.3.1. Overexpression and In Vivo Biotinylation

**Figure 3:**
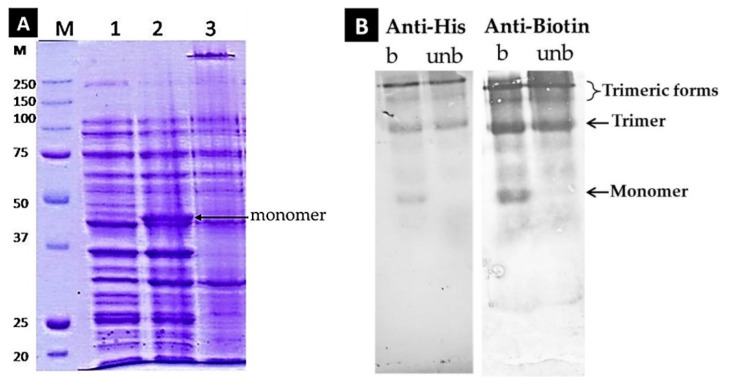
(**A**). Co-Overexpression of LHB in the BL21 DE3 strain of *E. coli* cells along with the pBirAcm plasmid that contains the gene of BirA biotin ligase. IPTG and biotin were added at the beginning of the 4 h incubation. Lane M: Markers; Lane 1: LHB before induction with IPTG/ boiled; Lane 2: LHB after induction with IPTG/ boiled; Lane 3: LHB after induction with IPTG/ unboiled. SDS-PAGE with a 7.5% gel was performed at RT. (**Β**) Western Blot analysis of the LHB protein for the detection of the His-tag and biotin moieties and the eventual presence of any degradation products. Penta Anti-His antibodies and Streptavidin-AP conjugate as “anti-biotin” probes were used. The LHB protein was examined in its boiled and unboiled state.

The LHB protein construct was engineered with a combination of functional groups as follows: the foldon domain at the C-terminus for enabling correct trimerization, a 6× His-tag for stabilization of the N-terminus and a 14-peptide immediately following the His-tag, that enables the attachment of a biotin molecule in vivo (Scheme 1E). The protein was overexpressed with the biotin ligase enzyme with the addition of IPTG and biotin for in vivo biotinylation. In Figure 3A at approximately 43 kDa in lane two, a distinct band after 4 h IPTG induction is observed, confirming the overexpression of the LHB construct. In lane three (unboiled sample), the band is absent and a slower migrating form is appearing, indicating that the protein must adopt a trimeric conformation.

#### 3.3.2. LHB Purification with Ni-NTA and Refolding after Dialysis

The cells were lysed with the French press method and, since the protein is located in the pellet, a further solubilization procedure with urea was followed. The supernatant after the denaturation step of inclusion bodies was loaded to a sepharose-Ni-NTA column. The LHB protein was eluted at increasing concentrations of imidazole between 50–150 mM. The protein elution fractions were further dialyzed against 20 mM Tris buffer pH: 9, NaCl 0.2 M and glycerol 5% in the cold room.

The folding and trimerization state were assessed as described in Figure S2. A western blot analysis was additionally performed to test the presence of the His-tag and of bound biotin by using the Penta anti-His antibody and Streptavidin–Alkaline phosphatase conjugate, respectively. As shown in Figure 3B, the LHB protein contains both of these moieties since they get recognized; furthermore, no smaller fragments and protein degradation products are present that bear these functional sites. 

#### 3.3.3. Chymotrypsin Digestion 

**Figure 4:**
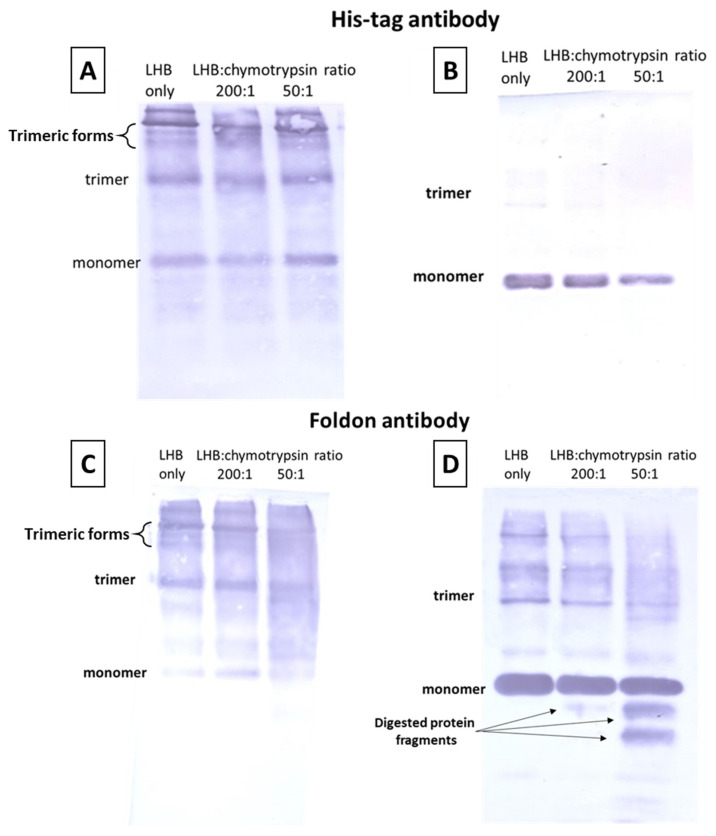
Chymotrypsin digestion experiments followed by electrophoresis and Western blot. The samples were electrophoresed and then probed with the His-tag antibody (**A**,**B**) and with the fibritin foldon antibody (**C**,**D**). In (**A**,**C**), unboiled samples were electrophoresed in the cold room; in (**B**,**D**) the samples were electrophoresed at RT after boiling at 100 °C for 5 min. Anti-His antibody detects the full-length protein and fragments that have an intact N-terminal end. Anti-foldon antibody detects the full-length protein and fragments that have an intact C-terminal end. LHB protein (0.2 mg/mL) was incubated with chymotrypsin for 10 min in various molar ratios (no chymotrypsin, 200:1, 50:1). Treated samples were subsequently frozen in 2% SDS loading buffer to quench the action of chymotrypsin.

The susceptibility of the protein LHB against chymotrypsin digestion was evaluated in order to detect the corresponding terminal end(s) being affected. LHB was incubated with chymotrypsin for 10 min in various molar ratios (no chymotrypsin, 200:1, 50:1). After 10 min digestion, the treated samples were immediately frozen following the addition of 2% SDS loading buffer to quench the action of chymotrypsin. The samples were electrophoresed in 7.5% acrylamide gel in the cold room in an unboiled state or at RT in a boiled state. After electrophoresis, the protein samples were transferred to a PVDF for Western blot analysis. The fibritin foldon wac antibody was used to detect the LHB full protein or any digested fragments that have intact their C-terminal end. Likewise, the presence of the His-tag tail implying an intact N-terminal end of the protein samples was detected with the Penta–His antibody. In Figure 4A, the SDS-PAGE electrophoresis of the unboiled samples in the cold room indicates that slower-migrating, trimeric forms are still recognized by the anti-His antibody; Figure 4B shows the results of the SDS-PAGE electrophoresis at RT after boiling the samples at 100 °C for 5 min. According to a densitometric analysis of the corresponding Coomassie-stained gels (Figure S5), the monomer band is significantly reduced after treatment with chymotrypsin at the 50:1 molar ratio in comparison to the untreated sample; moreover, there is no signal of anti-His antibody binding to any digested protein fragment migrating below the monomer band. This can be interpreted as follows: chymotrypsin starts to trim the LHB protein from the N-terminal end. As a result, partially digested trimeric forms still get recognized by the anti-His antibody, as one or two chains might still be in the full-length state. However, after boiling in (Figure 4B) the chains get dissociated and only the remaining full-length chains are recognized that migrate in the monomer position, whereas any digested chains are not recognized by the anti-His antibody, as they are missing the His tag, N-terminal part. In Figure 4C, SDS-PAGE electrophoresis of the unboiled samples in the cold room showed at the 50:1 ratio significant reduction of the trimeric LHB bands, detected by the multiple bands that create a smearing effect. Figure 4D depicts the results of SDS-PAGE electrophoresis at RT after boiling the samples at 100 °C for 5 min. At both 200:1 and 50:1 ratios, additional protein bands are detected underneath the monomer band indicating digestion into smaller fragments. The anti-foldon antibody recognizes these digested fragments meaning that the C-terminal end where the foldon motif is located is intact and unaffected. Therefore, the ensemble of the above data strongly suggests that the action of chymotrypsin starts from the N-terminal end which is more susceptible to digestion than the C-terminal end carrying the stable foldon trimerization motif.

#### 3.3.4. Conjugation of the Biotinylated LHB with the Enzyme Alkaline Phosphatase through the Streptavidin Molecule

**Figure 5:**
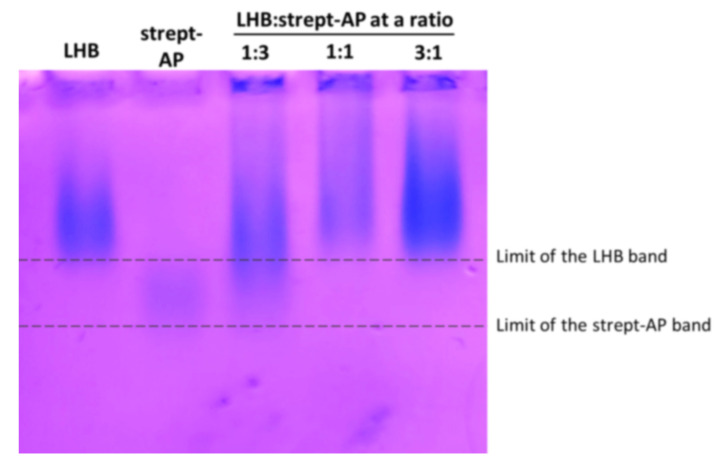
Native Agarose gel stained with Coomasie Blue for examining the binding of LHB-streptavidin-Alkaline Phosphatase (strept-AP) complex. Different ratios of LHB and strept-AP were used to evaluate the optimal binding ratio. Changes in the migration of the protein bands indicate efficient formation of the complex. The dashed lines delineate the migration boundaries of the free LHB and strept-AP and serve as reference for the better identification of the band shifting.

In order to determine the effectiveness of binding and the optimal ratio of LHB and streptavidin-Alkaline phosphatase to be used in delivery experiments, three different ratios (concentration: mg/mL) of LHB:strept-AP mixtures were incubated and analyzed into an agarose gel. The goal was to completely complex the streptavidin–Alkaline Phosphatase conjugate with the minimal LHB concentration possible. Free LHB and streptavidin-AP were used as controls to check the mobility of the unbound counterparts. LHB:strept-AP mixture at the ratio 1:3 resulted in partial binding of strept-AP as shown in Figure 5, since traces of the free strept-AP conjugate are still visible. Ratios 1:1 and 3:1 resulted in complete complexation of the AP conjugate, as indicated in the gel, since the strept-AP conjugate band is no longer visible within its migration zone (dashed line). Furthermore, the LHB band has slightly shifted higher and became more diffused, indicating that the complexation of strept-AP results in a bulkier moiety that migrates slower in the gel. Complexes with a ratio of 1:1 LHB:strept-AP were selected for further use in cell cultures since they contain the minimal amount of LHB. Additionally, before being used in the cell culture drug delivery experiments the LHB-strept-AP conjugate was incubated into a biotin-coated plate (Thermo-Scientific) in order to remove any streptavidin–Alkaline phosphatase molecules that remain non-conjugated. The same procedure was followed for determining the optimal binding ratio of LHB:strept-HRP and gave similar results (results not shown).

#### 3.3.5. Protein Nanorod Formation 

Adenovirus type 2 fiber folds into a stable trimeric protein conformation. The globular head located at the C-terminal end of the fiber acts both as a cell attachment organelle and trimerization motif, which enables the trimerization and self-assembly of the fiber protein into a nanorod conformation. In the LHB construct, the globular head was replaced with the fibritin foldon motif; moreover, the protein sequence starts at methionine 61 and not from methionine 1 as in the natural fiber. For using the LHB protein as a carrier, it is favorable to have a specific trimeric conformation and preferably to resemble the nanorod formation of the natural fiber. 

**Figure 6:**
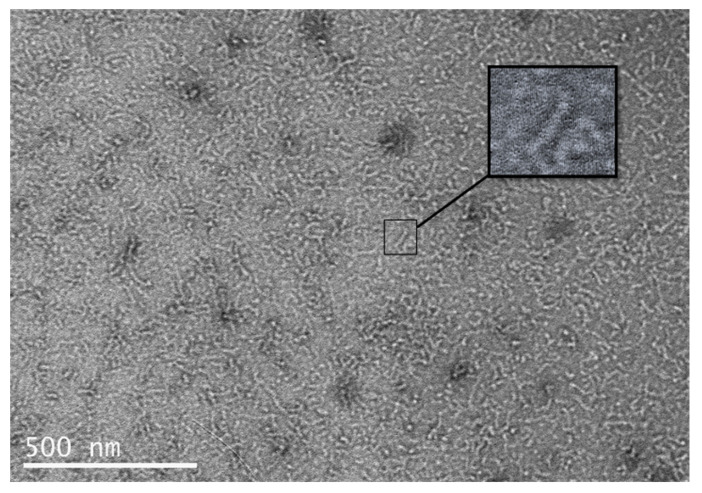
TEM picture of the LHB protein after purification, dialysis and negative staining. The insert points to one of the “straight” observed nanorods.

The native adenovirus fiber (residues 1–582), when observed in TEM after negative staining, displays a very characteristic “ball-and-rod” conformation, the ball corresponding to the globular head and the rod to the 22 repeats of the fibrous shaft [17]. Moreover, the TEM pictures very often display bent fibers, suggesting flexibility of the shaft segment [17]. The LHB construct was observed under Transmission Electron microscopy; the results depicted in Figure 6 show forms of thin nanorods of approximately 20 nm in length. Since the bulky globular head was replaced with the smaller foldon motif, the C-terminal “ball” is no longer distinguishable. Some of the fibrous rods appear “straight” and many appear bent, suggesting that the shaft retains flexibility.

#### 3.3.6. LHB Protein Exhibits High Thermal Stability 

**Figure 7:**
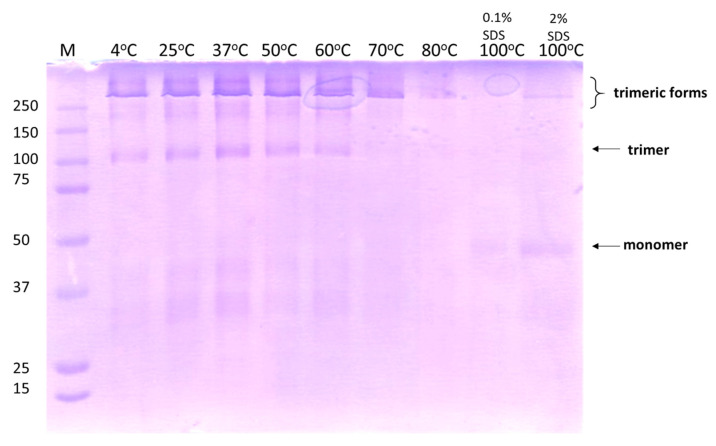
Thermostability of LHB after incubation for 30 min in each mentioned temperature. The 7.5% SDS gel was electrophoresed in the cold room to avoid any additional effects induced by the heating generated during the electrophoresis process. Gel analysis was conducted with Coomassie blue staining to detect the migration of the protein bands. In all samples after being heated, loading buffer containing 0.1% SDS was added. In the last sample being heated at 100 °C, loading buffer with 2% SDS was used for comparison. The arrows indicate the position of the protein bands corresponding to the trimeric native, folded state and to monomer unfolded state.

The “shortened” chimeric constructs carrying four shaft repeats connected to the foldon motif described previously [32] retained the stability and resistance to extreme conditions (SDS, temperature, denaturants) described for the full-length fiber. The chimeric protein LHB was tested for thermostability by incubating 0.4 mg/mL of the protein for 30 min under stepwise increasing temperatures. After incubation, 5 μL of ice-cold 3× concentrated loading buffer containing 0.1% SDS was added to each tube and the tubes were frozen at −20 °C for later processing. The resulting samples were analyzed in a 7.5% SDS gel in the cold room and visualized with Coomassie blue. In Figure 7, up to 70 °C only trimeric, slow-migrating states are present; upon further increasing the incubation temperature the higher migrating bands disappear and when the protein is heated at 100 °C, only the monomer state is visible. It is concluded that the LHB protein maintains high thermal stability.

### 3.4. Evaluation of the LHB Protein as a Protein/Nanoparticle Carrier to Cells 

#### 3.4.1. LHB Nanorods Can Spontaneously Internalize into Mammalian Cells with No Cytotoxic Effects

**Figure 8:**
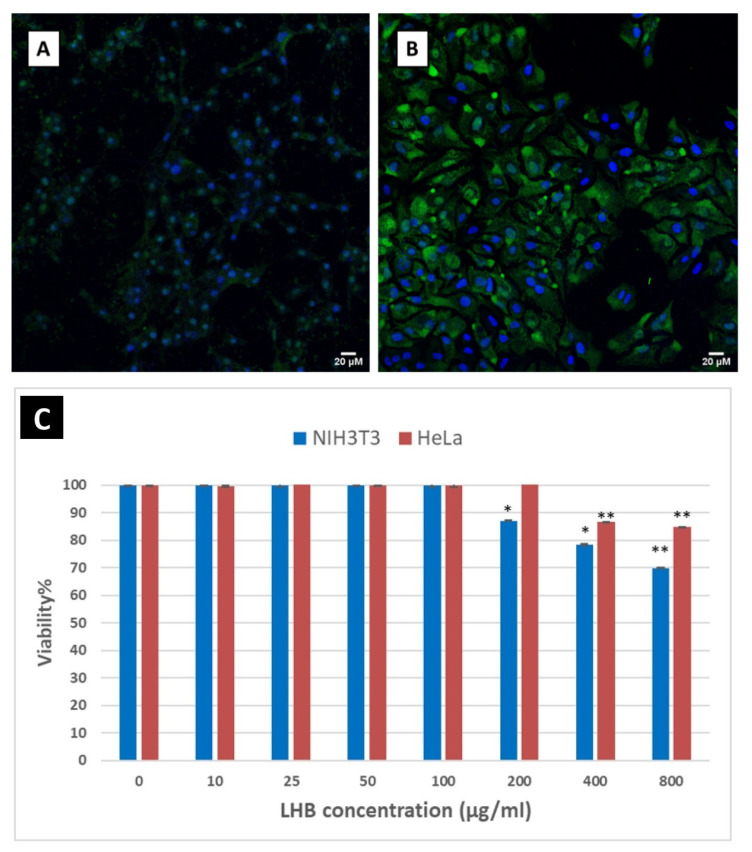
Intracellular delivery of LHB protein bound with NHS-Fluorescein (green) as a fluorescent tracker. (**A**) NIH3T3 cells and (**B**) HeLa cells. The nuclei are stained with DAPI (blue). Scale bar: 20 μm (**C**) MTT cell viability assay results of the LHB protein carrier incubated for 48 h in NIH3T3 and HeLa cells. Protein concentrations used varied from 10 to 800 µg/mL. Results are expressed as a percentage value of control cells cultured without the addition of the protein (control = 100%). Asterisks denote the significant difference in cell viability between the untreated cells and the transfected cells with the protein * is for *p* < 0.05 and ** for *p* < 0.01.

The Ad2 fibers with the globular head can interact with the CAR receptors in order to mediate docking with the cells. Replacing the head with another trimerization motif will ablate the interaction with this receptor. To this end, it is somewhat surprising that the knobless trimeric proteins are still able to internalize into the cells through other temperature-independent mechanisms and distribute to the cytoplasm and nucleus [30]. These alternative endocytic entry mechanisms might eliminate the constraining need to use the globular trimerization motif that specifically binds to CAR cell receptors. Moreover, it was previously reported that the isolated fiber protein in the absence of the penton base can enter the cells. The globular head by itself cannot enter the cells and accumulates in the CAR receptors located in the cell surface. This report suggested for the first time a potential role of the shaft domain in cell entry [16]. To determine whether the LHB protein can independently cross the cell membrane and access the cytosol and to additionally examine its eventual intracellular localization, immunostaining and confocal microscopy were performed in order to visualize NIH3T3 and HeLa cells following treatment with the protein. The amino groups of the LHB proteins were previously conjugated with the NHS-fluorescein. After dialysis to remove the excess of the free fluorescent molecule, LHB was incubated overnight with cells. As depicted in Figure 8A,B, the LHB protein effectively internalizes both cell lines and is detected in the cytosol.

While non-viral recombinant protein carriers for delivery are not associated with concerns compared to viral vectors, these applications do not come without potential adverse side effects. The most common one is the eventual cytotoxicity of the carriers towards the targeting mammalian cells. HeLa and NIH3T3 cell viability was studied in the presence of increasing concentrations of LHB protein by using the MTT assay. No significant cytotoxicity was detected after incubation for 48 h with LHB for both cell lines and only a decrease down to 70% viability was detected for NIH3T3 fibroblast cells after incubation with a very high concentration of LHB (800 μg/mL). Similar viability results were obtained for the HeLa cell line, with the lowest viability being 85% (Figure 8C).

#### 3.4.2. Efficient Transfer of the Fluorescent Streptavidin Gold Nanoparticles into the Cells by the LHB Protein Carrier Mediated by the Biotin–Streptavidin Bond 

**Figure 9:**
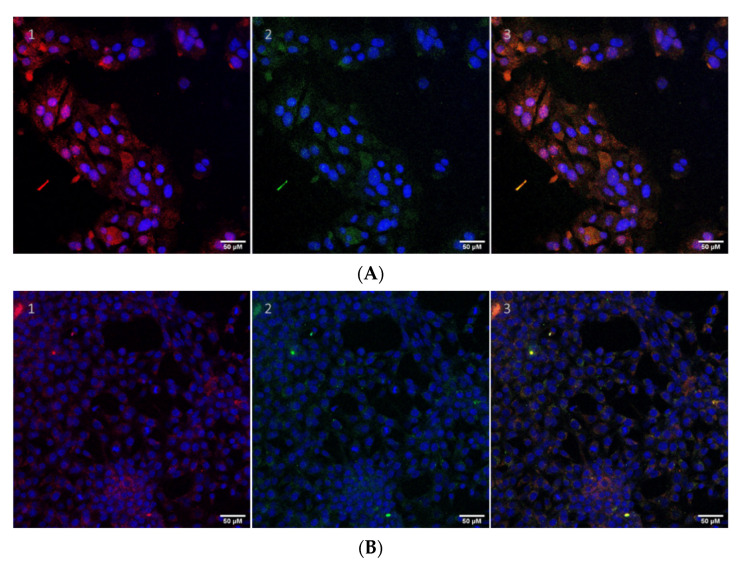
The LHB protein is attached to AlexaFluor^®^594 streptavidin Nanogold^®^ by the means of the biotin-streptavidin strong non-covalent bond. Cell internalization studies of the LHB protein-streptavidin gold NP conjugate in HeLa (**A**) and NIH3T3 (**B**) cells are shown. The protein construct is labeled with NHS-fluorescein (green) and further to the streptavidin-gold NP which contains an exposed Alexa595 dye (red). Cell nuclei are stained with DAPI (blue). Z-stack measurements of the cells were also obtained to ensure that the protein conjugates localize inside the cells at the same level. (**1**) Only the nuclei (blue) and the AlexaFluor594 (red) filters are applied in the confocal microscope. (**2**) Only the nuclei (blue) and the fluorescein (green) filters are active. (**3**) All filters are applied and the green and red signals are co-localized mergings into a yellow–orange color, indicating the effective transport of the intact complex. Scale bar: 50 μm.

Since the LHB protein can spontaneously internalize into the cell, the next step is to investigate its ability to act as a delivery agent that can deliver cargo molecules to the cell. LHB protein molecules labeled with NHS-fluorescein (green fluorescence) were incubated with the AlexaFluor^®^594 FluoroNanogold™-Streptavidin gold nanoparticles (Nanoprobes), which consist of streptavidin molecules attached to the 1.4 +/10 % nm diameter Nanogold^®^ particle and conjugated to AlexaFluor^®^ 594 dye (2–3 fluorophores/streptavidin molecule)–(red fluorescence)**.** Excess of streptavidin gold-coated nanoparticles was removed by incubation of the conjugate in a biotin-coated plate. Attachment of the fluorescein-conjugated LHB molecules to streptavidin–Alexa Fluor^®^ 594 FluoroNanogold nanoparticles would presumably lead to the fluorescent visualization and co-localization of both LHB protein (green fluorescence) and the nanoparticles (red fluorescence) by confocal microscopy in a human cell line (Figure 9A,B). The merged image (orange/yellow) indicates that the fluorescein (green) and AlexaFluor594 (red) signal are co-localized; therefore, the LHB protein acted as an effective carrier for the transportation of the streptavidin-gold NP into the cell and has the potential to act as a protein vector for cell internalization of nanoparticles.

#### 3.4.3. Localization of LHB Linked to Streptavidin–Alkaline Phosphatase Conjugate by BCIP-NBT Staining

**Figure 10:**
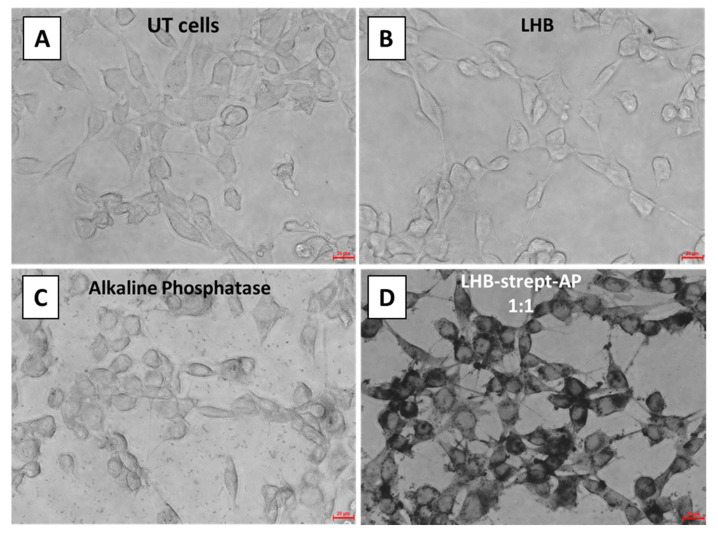
BCIP-NBT substrate staining of NIH-3T3 fibroblast cells after incubation at 37 °C. (**A**) untreated (UT) cells, (**B**) treatment with LHB only, (**C**) streptavidin linked with the alkaline phosphatase (AP) enzyme (**D**) streptavidin-AP conjugated with the LHB protein at a 1:1 ratio. Scale bar is 20 μm.

As verified above with fluorescence microscopy, the LHB protein alone can spontaneously internalize the cells without the means of a transfection method or reagent. In order to assess whether the LHB protein complexed to streptavidin-AP (Alkaline Phosphatase) conjugate mediates internalization of the enzyme, an internalization experiment was conducted on NIH3T3, and the enzyme activity was detected with BCIP-NBT chemiluminescence assays. The conjugate LHB-strept-AP was only tested in NIH3T3 cells since HeLa cells naturally express high amounts of Alkaline Phosphatase due to their tumoral nature. BCIP-NBT was used as a substrate for AP detection into the cells. As mentioned above, the reaction product has a blue color and is insoluble in water, therefore rendering the cells visible with a dark coloring where the product is located, allowing observation with an optical microscope. The results showed a significant difference of color intensity (black color) between NIH3T3 cells incubated with LHB-strept-AP compared to free alkaline phosphatase and LHB alone, suggesting that LHB indeed mediates internalization of the streptavidin-AP conjugate that maintains enzymatic activity (Figure 10).

#### 3.4.4. Localization of LHB Linked with HRP by DAB Staining and Measurement of HRP Activity by Luminol Detection

**Figure 11:**
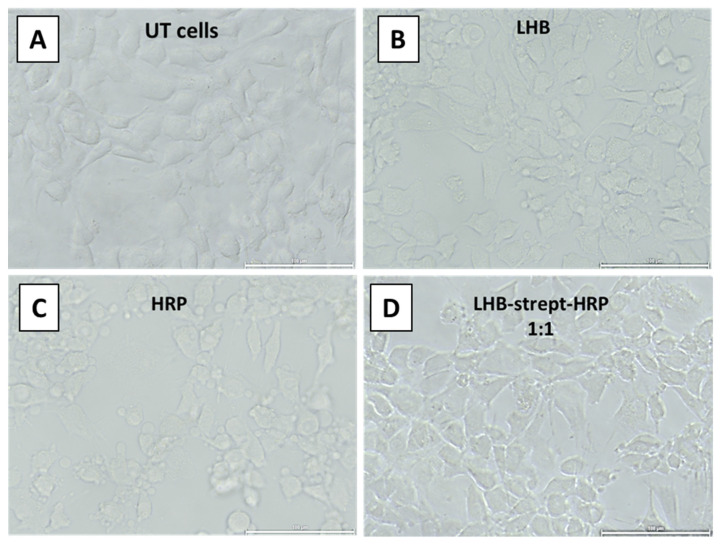
DAB staining of the NIH3T3 cells after incubation at 37 °C and observation under an inverted microscope. (**A**) no addition (Untreated cells), (**B**) addition of the LHB protein only (**C**) HRP-streptavidin conjugate only, (**D**) addition of the LHB-streptavidin-HRP complex at 1:1 ratio. Scale bar: 100 μm.

**Figure 12:**
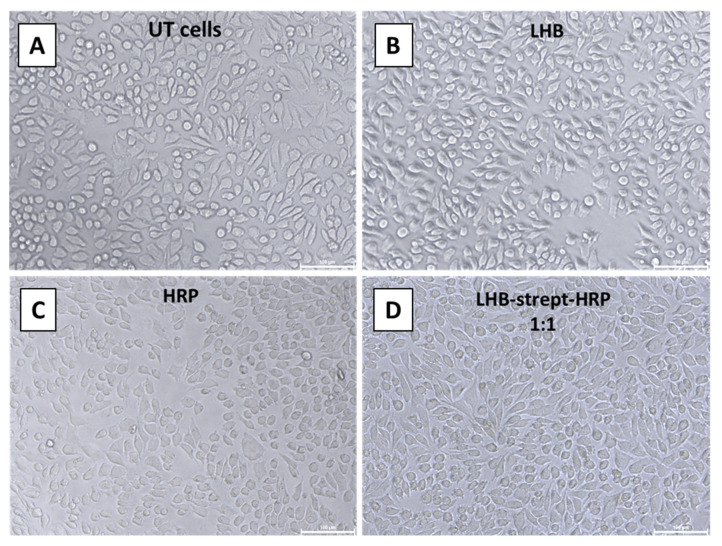
DAB staining of the HeLa cells after incubation at 37 °C and observation using an inverted microscope. (**A**) no addition (Untreated cells), (**B**) addition of the LHB protein only (**C**) HRP-streptavidin only, (**D**) addition of the LHB-streptavidin-HRP complex at 1:1 ratio. Scale bar: 100 μm.

The same methodology was used with a second model enzyme—horseradish peroxidase conjugated with streptavidin. In order to assess whether the LHB protein complexed to streptavidin-HRP (Horseradish Peroxidase) conjugate mediates internalization of the enzyme, an internalization experiment was conducted on both NIH3T3 and HeLa cells. Intracellular enzymatic activity was tested by 3,3′- diaminobenzidine (DAB) staining. The enzyme localization was determined after the addition and precipitation of DAB in the presence of H_2_O_2_. The visual detection of the developed brownish color diffused all over the cytoplasm area of the NIH3T3 or HeLa following treatment of the cells with the LHB-strept-HRP complex, providing evidence for the successful and intact transfer of the enzyme. Treated and untreated cells were observed directly by an inverted phase-contrast microscope. Cells incubated with free HRP-streptavidin and untreated cells served as control (Figure 11 and Figure 12).

**Figure 13:**
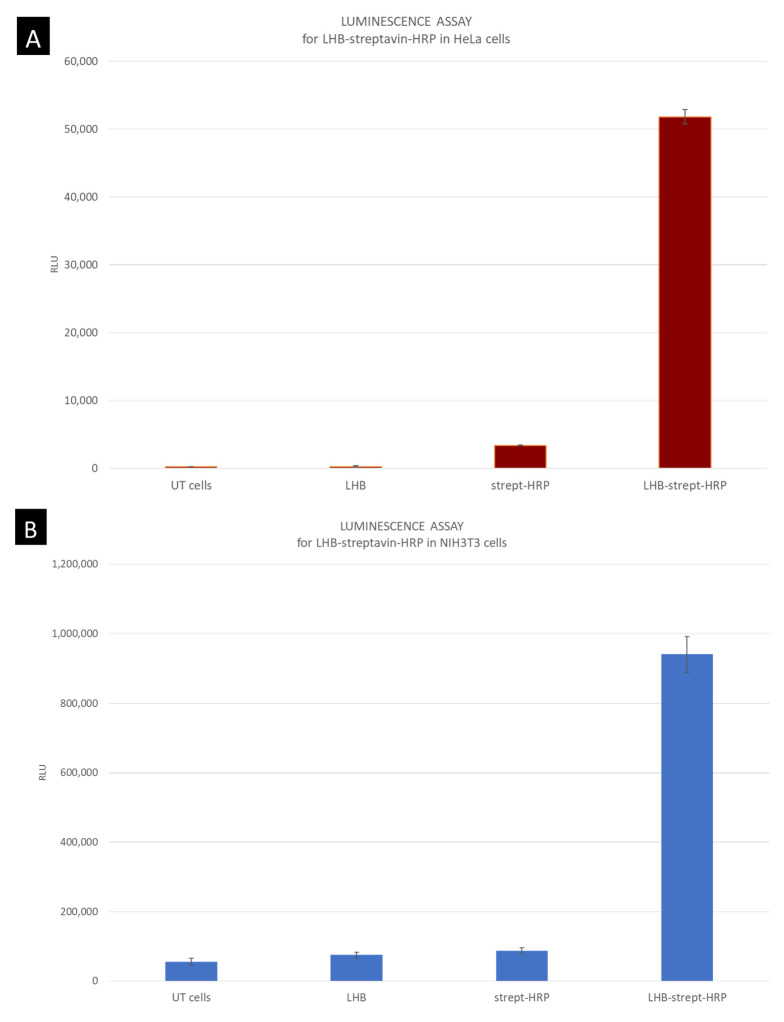
Measurement of the peroxidase light intensity levels produced after interaction with the substrate and H_2_O_2_. (**A**) HeLa epithelial cells and (**B**) fibroblasts NIH3T3. HeLa cells incubated with LHB-streptavidin-HRP conjugate have a 16× higher luminescence production in comparison to the streptavidin-HRP incubation. Luminescence for NIH3T3 cell line was 10× higher in cells treated with the LHB-streptavidin-HRP conjugate. Untreated cells and LHB were used also as negative controls.

For the quantification of the enzymatic activity of HRP transfer into the cells mediated by LHB, a more sensitive method was used for the detection of the peroxidase activity. By using the Supersignal West Pico PLUS Chemiluminescent substrate kit the light produced was measured in a luminometer. The results demonstrate a significant difference in light intensity produced when cells were incubated with the streptavidin conjugated peroxidase enzyme and when the conjugate was carried inside the cells as a complex with LHB. For both cell lines, the enzymatic activity measured was significantly higher when cells were incubated with the LHB bound streptavidin-HRP in comparison to streptavidin-HRP only. The difference in intensity is 10× higher for the NIH3T3 cells when treated with the LHB-HRP conjugate in comparison with the streptavidin-HRP conjugate and the intensity levels are 16× higher in HeLa cells treated in the same way (Figure 13A,B). The formation of the streptavidin-HRP complex with the LHB protein significantly increases the peroxidase access to the interior of the cells. Moreover, it is proposed that this binding and transfer do not interfere with the structure and activity of peroxidase. 

Taken together, the LHB carrier significantly enabled the internalization of active peroxidase molecules and subsequently led to higher light levels produced intracellularly. It can be therefore concluded that the LHB protein can act as a carrier and mediate the transfer of an intact enzyme to a cell line. The LHB protein cannot target a specific cell line since the fibritin foldon domain has no known cell receptor. By further modifying the protein sequence and by adding specific tissue targeting motifs, cell lines can be selectively targeted. 

## 4. Discussion

Fibrous nanostructured objects, including protein nanoneedles and self-assembling peptides, can be used as platforms for biomedical and nanotechnological applications. Elegant examples include protein nanoneedles [43,44,45] and amyloid-type or prion peptides [46,47,48]. These stable nanofibrous biomaterials targeted for such applications should not trigger cytotoxic responses. Moreover, they should adopt a well-defined conformation stable and resistant to harsh conditions. In this study, we designed and studied adenovirus protein fiber constructs for potential applications such as carriers for the delivery of protein and nanoparticle cargoes. We used as a basic structural framework the fibrous shaft segment of the adenovirus fiber protein comprising residues 61–392, connected to the fibritin foldon trimerization motif at the C-terminal end with or without the natural linker sequence (Asn-Lys-Asn-Asp-Asp-Lys-Gly-Ser) between them. These constructs [L] and [NoL] (B and C respectively in Scheme 1) were overexpressed in the form of inclusion bodies and subsequently were refolded into trimers; however, they were found to be partially digested during the overexpression/purification and/or refolding process. The addition of a 6× His-tag at the N-terminal end of the protein construct C leads to construct [LH] (D in Scheme 1) and improved the efficiency of the purification process through a one-step Ni-NTA affinity column and most importantly, stabilized the protein against proteolytic degradation during the overexpression/purification and/or refolding processes. As a next step, a fourteen-amino-acid biotinylation sequence was inserted immediately after the N-terminal, His-tagged end of the construct in order to enable the attachment of a biotin moiety in vivo. The protein construct [LHB] (E in Scheme 1) was overexpressed along with a biotin ligase enzyme (BirA ligase), and the successful attachment of the biotin molecule was verified with a streptavidin-Alkaline phosphatase conjugate in a Western blot. The [LHB] proteins were overexpressed in abundant quantities in *E. coli* and were subsequently studied in order to assess their structure and stability. They were found to self-assemble into a trimeric nanorod conformation as observed with TEM microscopy; furthermore, they were found to be thermally stable up to 70 °C. Thermostability is an important parameter to take into consideration when designing protein carriers for delivery purposes. Any delivery agent should be able to withstand the temperature of the human body or at least stay stable and not rapidly aggregate until the carrying therapeutic molecule reaches its target in the cell. In vitro digestion experiments with chymotrypsin were carried out; at high protease-to-protein ratios, the LHB proteins could be digested from their N-terminus. In an intracellular context, they would presumably release their N-terminally attached protein cargo into the cell cytoplasm. The biotinylation site added to the chimeric protein Linker-His-Biotin (LHB) enables the addition of a biotin molecule during the overexpression process into the bacterial cells and the subsequent in vivo biotinylation. Advantages that are associated with the in vivo biotinylation in contrast to the in vitro biotinylation are the uniform modification of the protein that excludes the use of intervening chemistry. Additionally, there is no need to separate modified products from unreacted ones and moreover, the purification of the ligase enzyme from the overexpressed protein is avoided. Although the LHB protein does not contain the CAR receptor binding motif located in the natural globular head of the fiber, it can internalize into cell lines such as HeLa and NIH3T3 fibroblasts cells. This confirms previous studies that reported internalization of the fiber protein in the absence of the virion and indicates that another currently undeciphered mechanism is allowing the internalization of the fiber; however, deciphering the exact internalization mechanism is beyond the scope of the present study. MTT assays showed no significant cytotoxicity of the LHB protein towards the aforementioned mammalian cell lines. As a next step, we sought to exploit the streptavidin-biotin strong non-covalent bond in order to bind and transfer biotinylated moieties into the cell. The protein LHB construct was able to transfer into mammalian cells (HeLa and NIH3T3 fibroblasts) a streptavidin-conjugated gold nanoparticle with an attached fluorophore. Most importantly, two streptavidin-conjugated model proteins, alkaline phosphate and horseradish peroxidase can be delivered in the cell cytoplasm by the LHB carrier and retain their enzymatically active form. 

Taken together, a series of constructs were fabricated to examine possible parameters for the design and engineering of an optimal molecule carrier. The optimal carrier chosen was the LHB protein, an N-terminally His tagged, in vivo biotinylated fibrous construct comprising the adenovirus fiber shaft residues 61–392 connected to the fibritin foldon motif. The experimental results show that LHB proteins are structurally stable, non-cytotoxic, and display functionalities that can be rationally engineered onto the initial protein structural framework. The work presented in this study aimed at establishing the proof-of-principle for the use of adenovirus fiber-based proteins as nanorods for the delivery of nanoparticles and model proteins; future experimental avenues would also include the exploration of the proteins used as therapeutic protein carriers. Of note, the shaft domain carries exposed loops including the extended loop comprising residues 346–355, offering the possibility of site-directed mutagenesis or sequence replacement for targeted anchoring. The display of heterologous viral epitopes towards vaccine development can also be envisaged, the most rational choice being the replacement of these extended loops on the surface of the LHB protein. 

The long-term prospects will be the development of multifunctional and modular fibrous nanorod platforms that can be tailored to the application at the sequence level. For example, they could target a specific type of cell or deliver a therapeutic molecule and an imaging nanoparticle at the same time. The present study reports a proof-of-principle for the development of a basic designable scaffold for future applications in nanomedicine. The viral origin of the recombinant protein prompted us to investigate cytotoxicity in two model cell lines, NIH3T3 and HeLa, with no significant cytotoxicity detected for protein concentrations ten times higher than the standard LHB concentrations used in the experiments. *E. coli* is the most common and widely accepted system for protein engineering and structural biology investigations. Nevertheless, for any future application destined for use in humans, the endotoxin effects associated with proteins produced in bacterial cultures have to be considered; as such, endotoxin-free expression systems, for example, *E. coli* strains [49] *Lactobacillus lactis* [50] and *Pichia pastoris* [51] could be considered. Moreover, besides the widely used mammalian cells, plant expression systems could be considered as well [52]. The proven thermostability of the constructs reported here guarantees safe delivery until any cargo reaches its target, and additionally, the thermostability of a carrier significantly lowers its handling and shipping costs. Last but not least, they can serve as protein-only platforms, attached or not to nanoparticles, that could display heterologous viral epitopes and specifically target immune cells towards the development of future vaccines.

## Supplementary Materials

The following are available online at https://www.mdpi.com/article/10.3390/biom12020308/s1, Methods: “Native” SDS-PAGE and trimerization assessment; Figure S1: Representation of the protein migration states in SDS gels; Figure S2: The role of SDS percentage in loading buffer and the gel running temperature in the protein conformational state; Figure S3: SDS-PAGE (7.5%) of the cell lysates and inclusion bodies washes. Figure S4: Purification of proteins with a Q-sepharose column. Figure S5: Densitometric analysis of the Coomassie gels used for blot studies following chymotrypsin digestion.

## Abbreviations

MoMuLV	Moloney Murine Leukemia Virus
PBS	Phosphate Buffer Saline
BCIP–NBT	BCIP [5-bromo-4-chloro-3-indolyl-phosphate]–NBT [nitro blue tetrazolium]
IPTG	Isopropyl β-d-1-thiogalactopyranoside
IB	Inclusion Body
DAB	3,3′-diaminobenzidine
