# Supplementary material for: Adenovirus Fibers as Ultra-Stable Vehicles for Intracellular Nanoparticle and Protein Delivery
Source: Biomolecules. 2022 Feb 15;12(2):308. doi: 10.3390/biom12020308 (PMC8869412; doi:10.3390/biom12020308)
Supplement: Supplementary file 1 [file biomolecules-12-00308-s001.zip › biomolecules-1526661-supplementary.pdf]

Adenovirus Fibers as Ultra-Stable Vehicles for Intracellular Nanoparticle and Protein Delivery

Chrysoula Kokotidou [1,2], Fani Tsitouroudi [2,†], Georgios Nistikakis [1,2], Marita Vasila [1], Katerina Papanikolopoulou [1,‡], Androniki Kretsovali [3] and Anna Mitraki [1,2,*]

[1] Department of Materials Science and Technology, University of Crete, 70013 Heraklion, Crete, Greece; chkokoti@hotmail.com (C.K.); nistikakisgiorgos@hotmail.com (G.N.); maritavsl@gmail.com (M.V.); papanikolopoulou@fleming.gr (K.P.)
[2] Institute of Electronic Structure and Laser (IESL), FORTH, 70013 Heraklion, Crete, Greece; ftsitouroudi@yahoo.com
[3] Institute of Molecular Biology and Biotechnology (IMBB), FORTH, 70013 Heraklion, Crete, Greece; kretsova@imbb.forth.gr
* Correspondence: mitraki@materials.uoc.gr
† Present address: UCB Celltech, Ajax building, 638 Ajax Avenue, Slough SL1 4BG, UK.
‡ Present address: Biomedical Sciences Research Centre 'Alexander Fleming', 34 Fleming Str., 16672 Vari, Attica, Greece.

Citation: Kokotidou, C.; Tsitouroudi, F.; Nistikakis, G.; Vasila, M.; Papanikolopoulou, K.; Kretsovali, A.; Mitraki, A. Adenovirus Fibers as Ultra-Stable Vehicles for Intracellular Nanoparticle and Protein Delivery. *Biomolecules* **2022**, *12*, x. https://doi.org/10.3390/xxxxx

Academic Editor: Maria Teresa Sciortino

Received: 10 December 2021
Accepted: 12 February 2022
Published: 15 February 2022

Publisher's Note: MDPI stays neutral with regard to jurisdictional claims in published maps and institutional affiliations.

Copyright: © 2022 by the authors. Licensee MDPI, Basel, Switzerland. This article is an open access article distributed under the terms and conditions of the Creative Commons Attribution (CC BY) license (https://creativecommons.org/licenses/by/4.0/).

Supplementary Information

Method: "Native" SDS-PAGE and trimerization assessment

The adenovirus fiber protein is resistant to SDS, namely dissociates into monomers only after boiling in 2% SDS [22]. When deposited in SDS-PAGE gels without boiling and the electrophoresis is carried out at 4 °C, the native protein does not migrate as an SDS-polypeptide chain complex, but instead remains trimeric and migrates at an apparent high molecular mass position. This resistance to SDS allows easy distinction of the native form of the protein from any misfolded or partially folded forms that get denatured by SDS and migrate in the monomer position. Moreover, any trimeric, partially unfolded forms migrate slower than the native trimer, since they are more expanded (the so-called "umbrella" effect) as depicted in **Figure S1**, see also [22]. Therefore, in order to detect any trimeric, non-denatured or partially denatured forms, samples are not boiled and the electrophoresis is carried out at 4 °C using 0.1% SDS in the loading buffer. We hereinafter refer to this protocol as "Native SDS-PAGE". Specifically, the protein trimerization state was assessed at both 4 °C and 25 °C. LHB protein (10 μL) at a concentration of 0.4mg/ml was boiled at 100°C for 5min by adding 5 μL of either 0.1% or 2% SDS sample buffer. An unboiled protein sample of the same concentration was also mixed with 0.1% or with 2% SDS sample buffer. The set of these four different combinations was electrophoresed in a 7.5% acrylamide gel in the cold room at 4 °C or at room temperature at 25 °C **(Figure S2).** The protein bands were visualized with Coomassie Blue staining.

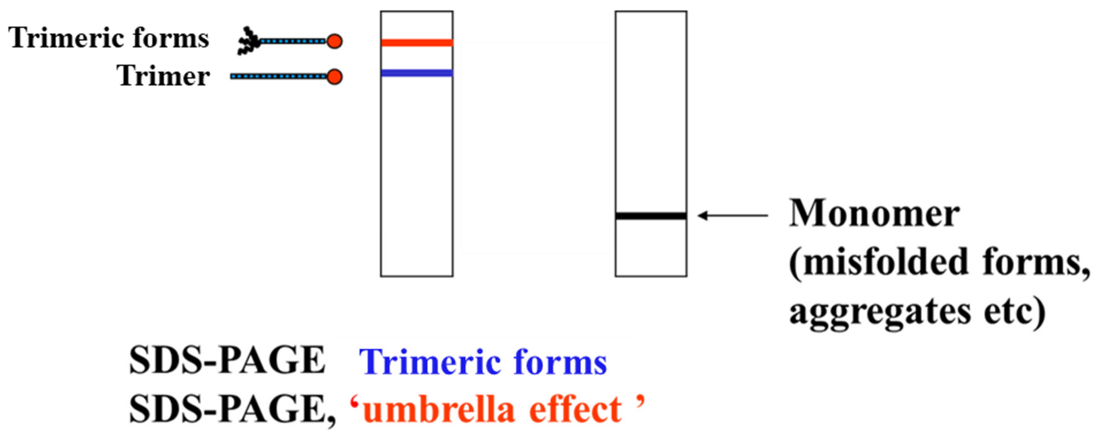

Figure S1. Representation of the protein migration on SDS-PAGE gel depending on its folding and trimerization state. The term "umbrella effect" refers to partially unfolded but still trimeric states (red band) that migrate slower than the native trimer (blue band), due to the retardation caused by the partial "opening" of the molecule. Of note, the higher molecular weight bands that are observed are in part induced in situ during the gel running process and do not entirely reflect the state of the protein in solution. This is due to fact that the protein encounters mild denaturing conditions, i.e., presence of SDS in the loading buffer and 0.1% SDS in the running buffer even if the samples are not subjected to boiling.

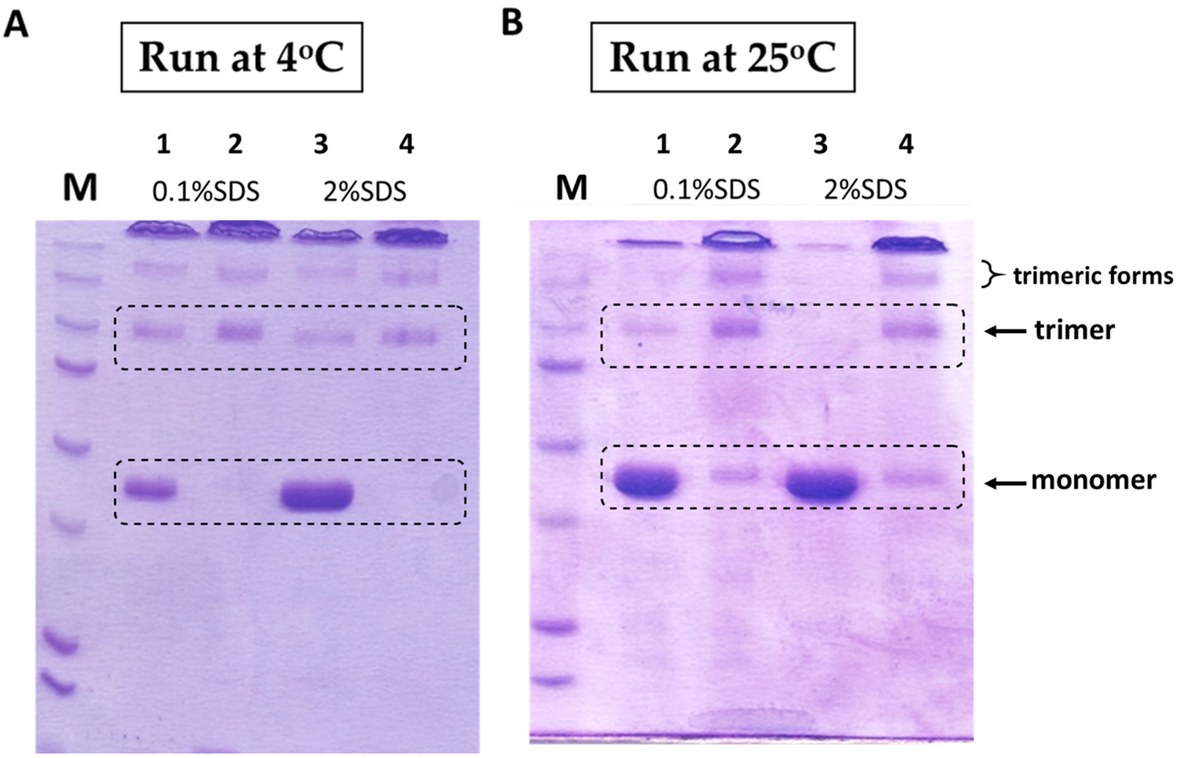

Figure S2. The role of SDS percentage in loading buffer and the gel running temperature in the protein conformational state. SDS-PAGE of LHB (**A**) in a cold room and (**B**) in room temperature. Lanes for both gels: M: Marker; 1: boiled with 0.1%SDS loading buffer (LB); 2: unboiled with 0.1% SDS LB; 3: boiled with 2% SDS LB; 4: boiled with 2% SDS LB. A 7.5% gel was used.

In **Figure S2A,** lanes 1 and 3, at cold SDS-PAGE running conditions and when the protein was boiled either in 0.1% or 2% SDS loading buffer, a trimer band is still visible along with higher migrating trimeric bans as explained in figure S1. A possible explanation might be that a certain percentage of the protein was not totally unfolded when heated at 100°C and due to the cold analysis conditions (which could not induce further unfolding) could be observed in the trimeric conformation. Alternatively, there is the possibility that although initially unfolded when boiled, the protein might be able to refold in the gel due to the low temperature running conditions. In the non-boiled samples, no monomeric bands are observed for both SDS loading buffer concentrations. For the gel run at room temperature **(Figure S2B),** in 0.1%SDS loading buffer (lane 1) a certain amount of the trimer band is observed in the boiled sample, in contrast to the sample boiled in 2% SDS (lane 3) where there are no visible traces of the protein trimer, the total amount being denatured in the monomeric state. In the non-boiled samples at both SDS loading buffer concentrations, a certain amount of monomer is observed, presumably due to in-gel heat denaturation (lanes 2 and 4). Therefore, the optimal conditions for assessing the trimeric state are to run the non-boiled samples in the cold room in 0.1 % SDS, whereas in order to assess the fully denatured, monomeric state, the boiled samples must be run at RT after boiling in 2% SDS.

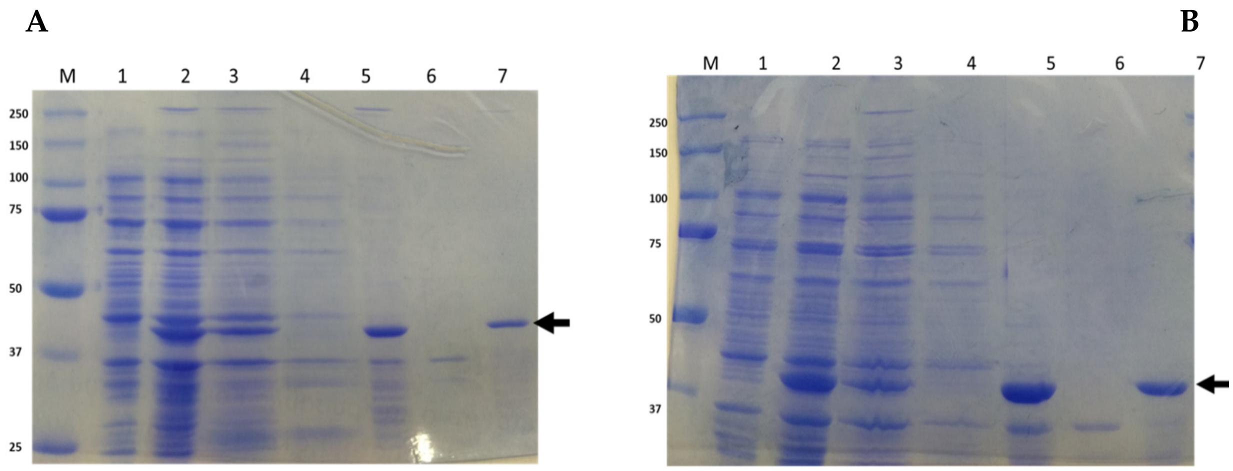

Figure S3. SDS-PAGE (7.5%) of the cell lysates and inclusion bodies washes. After cell lysis the proteins are located in the pellet fraction, indicating that they aggregated into inclusion bodies. (**A**) Linker protein (L) and (**B**) No Linker (NoL) protein. **Lanes** M: protein markers; 1: 0h before IPTG induction; 2: expressed for 4 h after IPTG induction; 3: protein lysate; 4: supernatant after centrifugation of the lysate; 5: pellet after centrifugation of the lysate; 6: supernatant after pellet washes; 7: pellet after further washes. All samples were heated at 100 °C for 5min with loading buffer containing 2% SDS. The arrows point to the monomer position.

A B

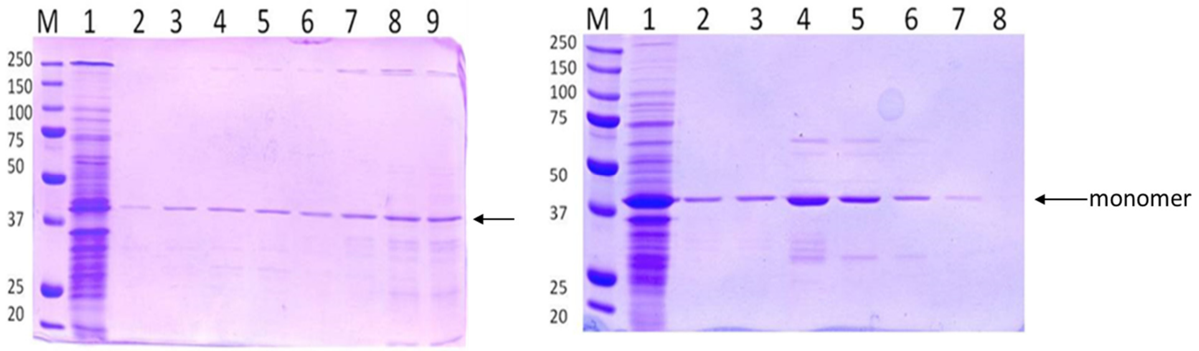

Figure S4. (**A**) **Purification of protein [L] with Q-sepharose column**. Elution of the protein with increasing concentrations of NaCl. The protein was eluted at 50-100mM NaCl. **Lanes**: M: protein markers, 1: Supernatant of the protein after cell lysis with urea 6M. **2–5**: elution fractions of the protein at 50mM NaCl. **6–9**: elution fractions of the protein at 100mM NaCl. (**B**) **Purification of protein [NoL] with Q-sepharose column**. Elution of the protein with increasing concentrations of NaCl. The protein was eluted at 50-100mM NaCl. Lanes: **M**: protein markers, **1**: Supernatant of the protein after cell lysis with urea 6M. **2–8**: elution fractions of the protein at 100mM NaCl. The arrows point to the monomer position.

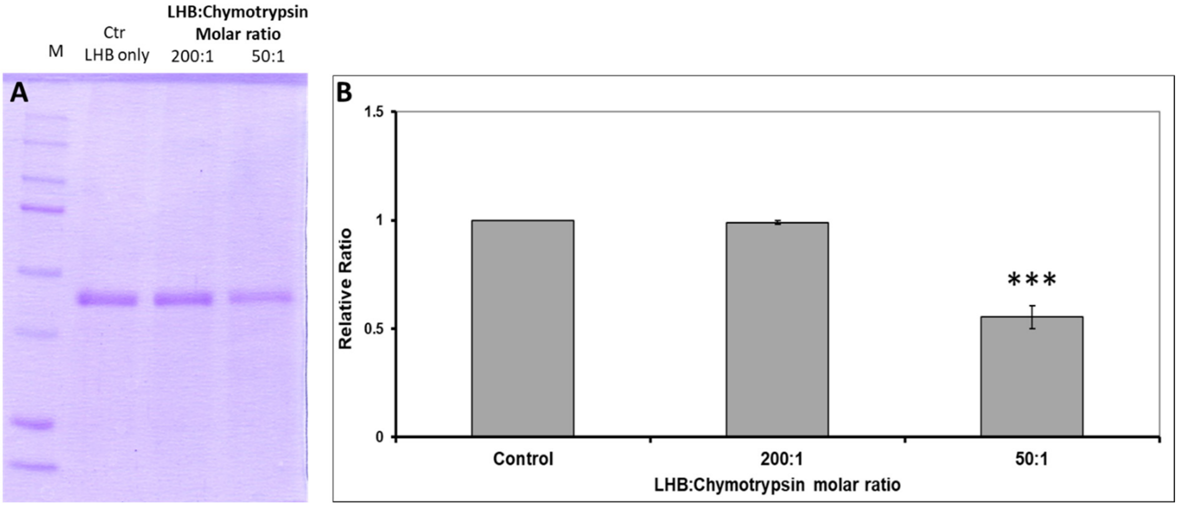

Figure S5. Densitometric analysis of the Coomassie gels used for blot studies following chymotrypsin digestion. (**A**) LHB protein digestion by the chymotrypsin enzyme in two different LHB to Chymotrypsin molar ratios (200:1 and 50:1). After digestion the samples were boiled and electrophoresed in a 7.5% SDS-PAGE gel. M: Molecular mass markers. (**B**) Signals were quantified by densitometry with the ImageJ program. Levels of the protein are shown as a ratio relative to their respective level in untreated control samples, which is set to 1. Results were plotted as means + SEM from four independent experiments. The data were analyzed by standard parametric statistics (Student's *t*-tests). Stars indicate significant differences ($p < 0.0001$) from the untreated control.
